# Integrated biostratigraphy of the Maastrichtian-Danian sequence in the southern Tethys with insights into paleoenvironmental implications

**DOI:** 10.1038/s41598-025-24036-1

**Published:** 2025-11-04

**Authors:** Ramadan M. El-Kahawy, Aya Raafat

**Affiliations:** Geology Department, Faculty of Science, Cairo University, Giza, 12613 Giza Egypt

**Keywords:** Nannofossil, Dinoflagellate cysts, Biostratigraphy, Paleoclimate, Paleoproductivity, Maastrichtian, Egypt, Palaeoceanography, Palaeoclimate

## Abstract

The Cretaceous-Paleogene (K/Pg) sequence offers a global perception of the paleoecological evolution in the southern Tethys realm. High-resolution calcareous nannofossil and dinoflagellate cyst analyses were conducted on subsurface Maastrichtian-Danian deposits in the Western Desert of Egypt, to characterize the paleo-conditions. The calcareous nannofossil analysis retrieved four main biostratigraphic zones (UC18-UC20, and NP4). A hiatus at the K/Pg boundary resulted in absence of most Danian nannofossil zones (NP1-NP3). The palynofacies assemblages discriminated three intervals of varying oxygen levels: dysoxic, proximal suboxic-anoxic shelf, and distal suboxic-anoxic basin incorporated by oligo-eutrophic conditions. The species diversity and relative abundance patterns of sensitive nannofossil indicators during this interval are attributed to significant climatic shifts. The surface waters during the Maastrichtian experienced a general cooling trend, interrupted by two warming episodes that may be correlated with the Deccan traps’ two phases (1&2). The biotic productivity indices and geochemical proxy (P, Sr/Al& P/Al) showed that during the earliest Maastrichtian high productivity prevailed, whereas during middle and late Maastrichtian a fluctuating pattern appeared. Statistically, multivariate techniques discriminated the retrieved taxa into five groups, each characterizing a depositional environment and climatic trend. Further, the palynomorphs analysis classified the Maastrichtian-Danian sequence into three offshore environments. A more refined understanding of climatic upheavals is essential for future studies to assess their potential role in driving biotic changes across the K/Pg boundary.

**Supplementary Information:**

The online version contains supplementary material available at 10.1038/s41598-025-24036-1.

## Introduction

The Maastrichtian stage, spanning 6.1 Ma (72.1–66.0 Ma^1^;, was marked by significant global climatic shifts that culminated in one of the most extensive mass extinction events at the Cretaceous/Paleogene (K/Pg) boundary^2^. The environmental and climatic conditions, which likely played a pivotal role in the mechanisms driving the K/Pg mass extinction, warrant greater efforts to understand this global biotic crisis^3–6^. The Late Cretaceous paleotemperature, particularly Maastrichtian, was reconstructed using an integrated TEX_86_, and δ^18^O_pl_, suggesting a cooling trend of the low-latitude sites^7^. However, Peral et al.^8^ conducted clumped isotope analyses on planktonic and benthic foraminifera from the Maastrichtian Chalk Sea in Poland, highlighting the warm conditions and the influence of Tethyan water incursions on bottom water. Li and Keller^9^, and Abramovich et al.^10^ have conducted studies that suggest the Maastrichtian stage was characterized by exceptional climatic instability, which led to two warming episodes^11,12^. The older one occurred during the mid-Maastrichtian, when the surface and bottom waters warmed from 2 to 3 °C^13^. The other episode represents the latest Maastrichtian warming event (LMWE), which is globally documented and precedes the K/Pg boundary and occurred between 450 and 100 kyr^14–17^. According to Barnet et al.^11^, this event is evidenced by a global bottom water temperature spike of 2.5–5 °C. Keller et al.^18^ proposed that releasing vast quantities of CO_2_ and SO_2_ from Deccan volcanism led to the mass extinction at the end of the Maastrichtian, coupled with ocean acidification. These CO_2_ emissions resulted in a 4 °C increase in global ocean temperatures^13^ and a 4 °C rise on land^11,19^. Furthermore, Woelders et al.^17^ estimated the LMWE warming of 3.9 ± 1.1 °C based on integrated multi-proxy data of benthic and planktic Mg/Ca, benthic *δ*^18^O, and TEX_86_ from the Bass River (New Jersey). Li et al.^20^ reconstructed a high-resolution record of mercury (Hg) spikes that coincided with the LMWE and aligned with the negative carbon isotope excursion, reflecting a period of global warming peaks likely driven by pulsed volcanic eruptions.

It is noteworthy that a high diversity of green algae (i.e., charophytes) in response to LMWE has been recorded from the Songliao Basin (Northeast China)^21^, along with an acme of tropical calcareous nannofossil in the Atlantic and Pacific oceans^14^, emphasizing the global extent of the LMWE and its profound impact on both terrestrial and marine biota. In contrast, the planktonic foraminifera responded negatively to LMWE via increasing relative abundance of the opportunistic taxa and the Lilliput (dwarfing) effect^22^. The dinoflagellate cyst response to the K/Pg before, across, and post-event showed fluctuating behavior, reflecting complex climatic and environmental shifts associated with this mass extinction interval. In the El-Kef^23,24^ and Elles^25^ sections along the Tunisian shelf, the dinoflagellates’ response was unscathed and exhibited during the latest Maastrichtian a high diversity and abundance of warm water taxa. On the other hand, across the K/Pg boundary, significant fluctuations and abrupt shifts in species composition have been observed, denoting the initiation of a cooling pulse and sea-level fall. By the early Danian, multi-spikes of higher-latitude dinocyst incursions signify replicated cooling episodes and persistent climatic environmental instability. In Turkey, during the latest Maastrichtian, a high diversity dominated by a pronounced spike of *Manumiella druggii* has been observed, followed by first occurrences of early Danian taxa, including *Senoniasphaera inornata*, and *Damassadinium californicum*^26^. Furthermore, in the Okçular section (NW Turkey), the species diversity showed little variation across the boundary, with dinocyst assemblages consistently dominated by the Spiniferites complex (gonyaulacoid) before and after the event^27^. In Egypt, a highly diverse and well-preserved dinocyst assemblage was retrieved in Dababiya Borehole during the latest Maastrichtian with boundary markers, including the (LOs) of *Cannosphaeropsis utinensis*, *Alisogymnium euclaense*, *Dinogymnium* spp., and *Pterodinium cretaceum*, and followed by abrupt turnover at the K/Pg boundary^28^.

The Maastrichtian-Danian climatic trend has been recorded across different paleolatitudes using calcareous nannofossil assemblages^29–32^. However, a detailed pattern of climatic perturbation is less documented in the sedimentary archive of the Egyptian territories in the low-latitude region. Furthermore, the Late Cretaceous dinoflagellate cyst ascertained their significance in climatic reconstruction using sensitive indicator taxa^25,33^. The utilization of dinoflagellates as a biotic proxy for diagnosing paleoclimatic conditions during the Maastrichtian-Danian interval in the Western Desert of Egypt, specifically at Farafra Oasis, remains scant. Although few published subsurface works in the Western Desert, particularly Farafra Oasis, focusing on the paleoenvironmental changes occurring below and above the K/Pg boundary, most calcareous nannofossil and palynomorphs (e.g., dinoflagellate cysts) studies on the Maastrichtian-Danian sediments in Egypt are mainly dedicated to biostratigraphy^34,35^. Few integrated studies have been conducted on either surface or subsurface Maastrichtian-Danian deposits^36^. This highlights a research gap in understanding the response of nannofossil and dinoflagellate cyst assemblages to climatic shifts and paleoproductivity changes across the K/Pg interval. Meanwhile, the study area lacks integrated work based on biotic and abiotic proxies, thus examining the nannofossils and palynomorphs enhances our understanding of the region’s biostratigraphy and palaeoecological conditions.

The main goals of the present work are (1) to build-up a detailed and robust biostratigraphic framework for the Maastrichtian to Danian periods in the area of study via utilizing nannofossil and palynomorph marker taxa, (2) to address and emphasize the response of the nannofossil and dinoflagellate cyst populations to the Maastrichtian/Danian climate and environmental changes using quantitative analysis of biotic and a biotic proxies, (3) to reconstruct the prevailed paleoenvironmental conditions and ecological dynamics in the Farafra Oasis using palynomorph and nannofossil data. Therefore, in this study, we performed micropaleontological and geochemical analyses on the collected sediment samples from the B-24 borehole.

## Geologic setting and lithostratigraphy

**Fig. 1:**
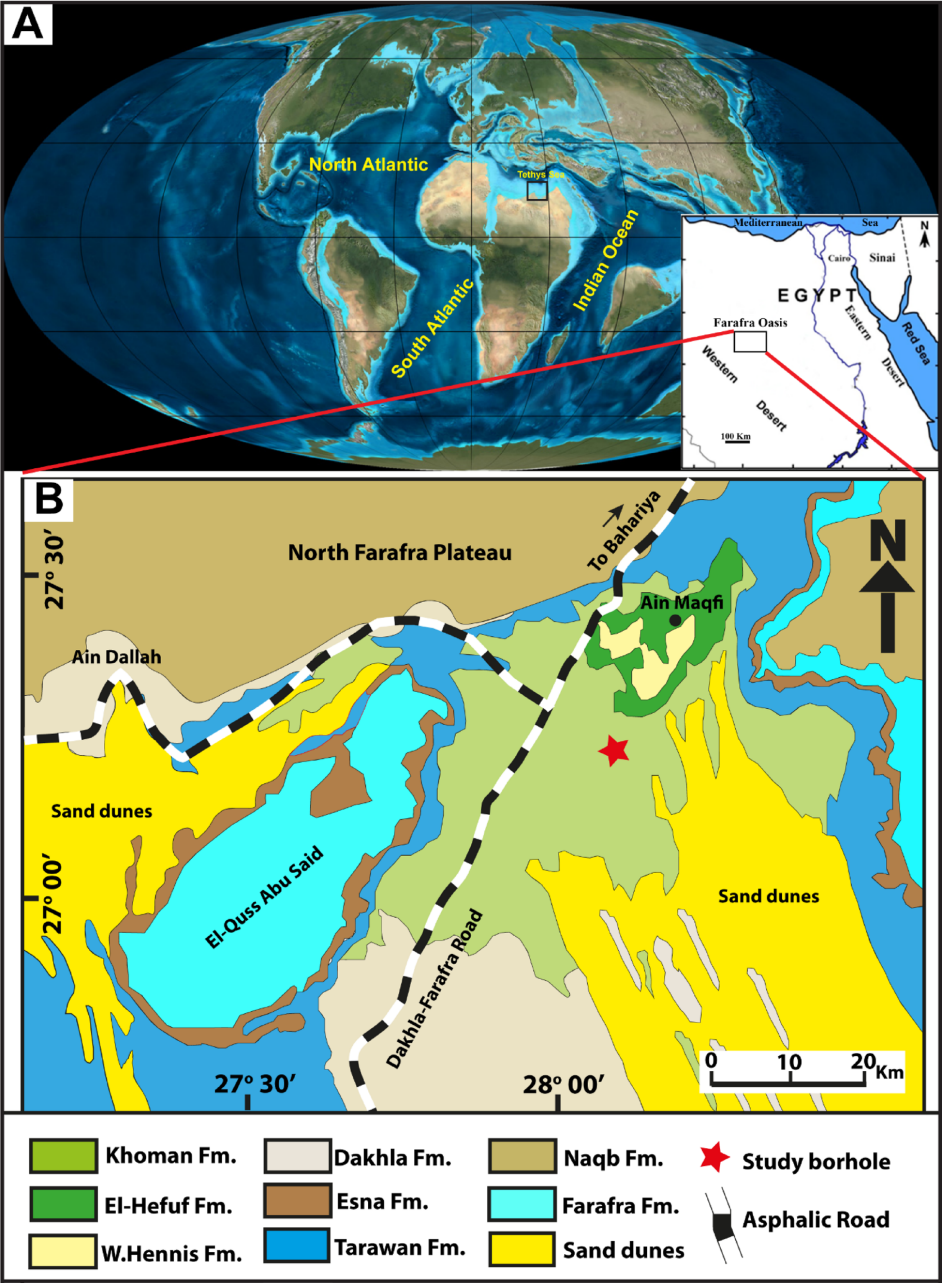
**A**. Paleogeographic map for the Late Cretatceous-Early Paleogene (Extracted from time scale creator V.8., https://timescalecreator.org/index/index.php), **B.** Geologic map for the investigated rock units in the Farafra Oasis (modified after^150^.

In Egypt, the Farafra Oasis (Fig. 1) lies in the southern part of the Neo-Tethys shelf and occupies the central part of the Egyptian Western Desert^37^. During the Cretaceous, the Farafra Oasis was subjected to the tectonic influence of the Syrian Arc system, which was responsible for the paleo-highs and paleo-lows in the northern Sinai and the Baharyia Oasis, which continued up to the late Eocene^38^. A major marine transgression prevailed along with the lateral compression of the Syrian Arc, producing distinct carbonate facies reflecting changes from clastic to non-clastic domain^39^. Consequently, the sea-level variations have a significant impact on the formation of the Bahariya-Farafra platform during the Late Cretaceous^40–42^. Throughout the Cenomanian, the Bahariya-Farafra platform drifted on a passive continental margin^41,43^, while in the Turonian-Santonian, the northern part of the Farafra was tectonically uplifted, and the southern part was subsiding^41,43^. Up to the Maastrichtian, the northern part of the Farafra Oasis was submerged and covered by marine setting^43^. In the Paleocene, the shales of the Dakhla, chalky limestones of the Tarawan formations, and the overlying Esna Shale were deposited^44^.

Stratigraphically, the Maastrichtian chalk and chalky limestone form the floor of the Farafra Depression, which is partly covered with wind-blown sand dunes and playas of the Great Sand Sea. The Dakhla Shale (Maastrichtian) is outcropped on the southern sector of the depression and laterally wedged into the Maastrichtian Khoman Chalk in the central and northern parts of the oasis^44^. These facies changes are associated with the syn-sedimentary tectonism of the Syrian Arc Orogeny^45^. The scarps of the depression consist of the Paleocene Tarawan Formation overlain by the Esna Shale (Paleocene-lower Eocene) and the lower Eocene Farafra Limestones^44^. The north-to-south transect of the Western Desert exhibits lithological variations, wherein the Farafra area (north), a carbonate rock unit dominated the Upper Cretaceous, while to the south, the clastic rock units of argillaceous materials were widespread^46^.

**Fig. 2:**
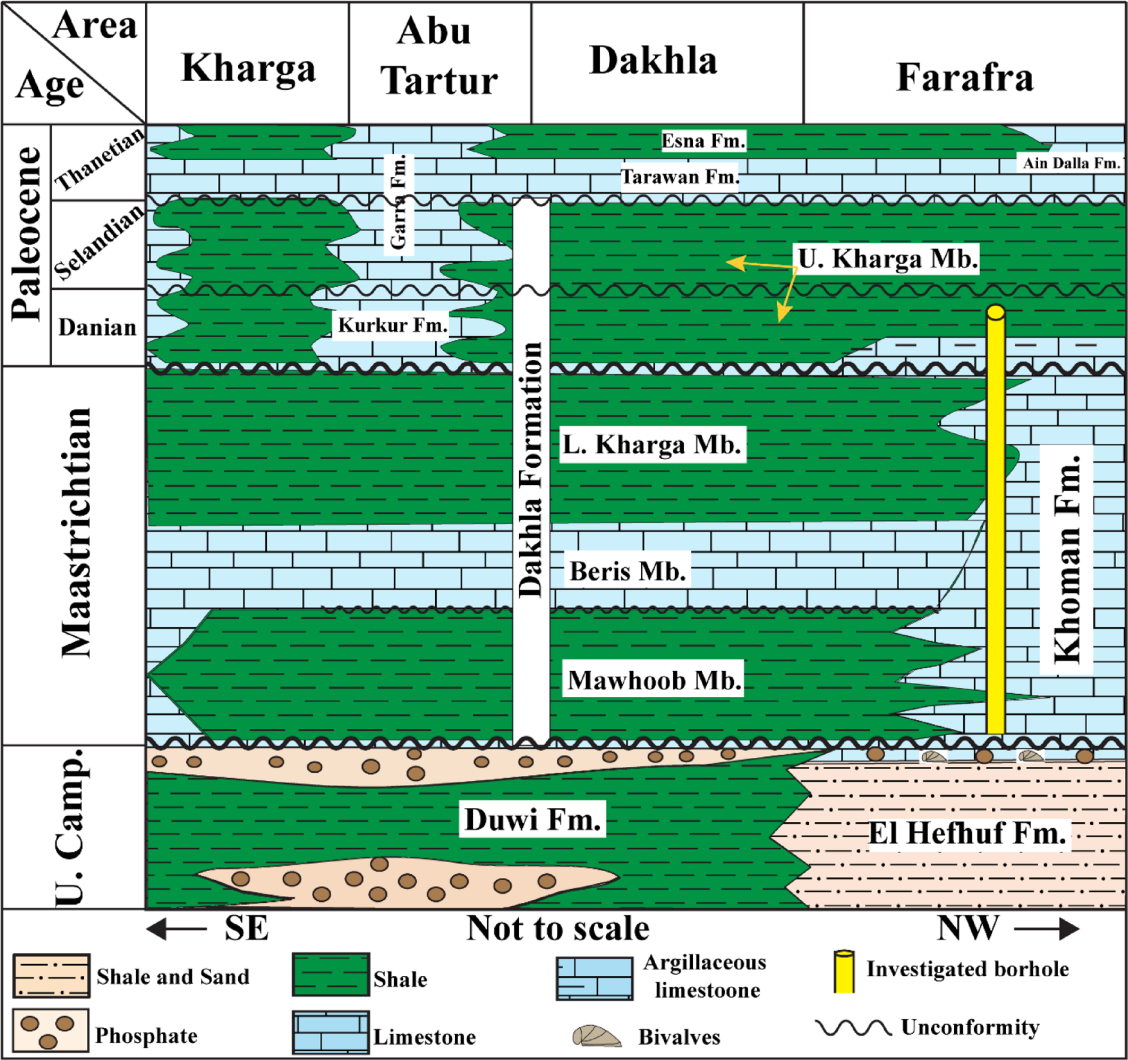
Lithostratigraphic model for the facies variabilities from the Farafra across the Kharga oases (modified after^46^.

The Upper Cretaceous (Maastrichtian)- lower Paleocene (Danian) sedimentary sequences in the Farafra Oasis, Western Desert of Egypt, exhibit progressive lithological variations classified into two formations: Khoman (at the base) and Dakhla (at the top). The basal Paleocene Dakhla Formation unconformably superimposes the Maastrichtian Khoman Formation (Chalk) (Fig. 2) and will be described below from older to younger:

Kerdany^47^ introduced the Khoman Formation to delineate the open marine carbonate facies at the Ain Khoman, located south of the Bahariya Oasis in the Western Desert of Egypt. The Khoman Fm., which is approximately 120 m thick in the investigated borehole (B-24) and consists of snow-white limestones, where it is the main rock unit in the present study (Fig. 2). To the south, it is intervened by numerous intercalations of shale sediments, which is attributed to the inter-tonguing between the Khoman Fm. (chalky limestone) in the Farafra Oasis and the Dakhla Formation (shale) in the Abu Minqar. The Dakhla Formation consists mainly of nodular to fissile calcareous shales interspersed with thin layers of argillaceous limestone^48^. It has been documented to be 10 m thick in the investigated borehole (Fig. 2). The investigated borehole (B-24) is located to the north of the transition zone between carbonate sediments (chalky limestone) and siliciclastic sediments (shale).

## Materials and methods

Fifty-one sediment samples were obtained from PetroServices drilling company and cover a thick sequence of approximately 130 m of the borehole (B-24) spanning the Upper Cretaceous -Lower Paleogene Khoman to Dakhla formations (Fig. 1). The borehole investigated was acquired from the Farafra Oasis, Northern Western Desert, Egypt, which displayed variegated lithological characteristics from chalky carbonate to argillaceous shales (Fig. 2). The adopted micropaleontological proxy involves quantitative analysis for both calcareous nannofossils and palynomorphs.

**Fig. 3:**
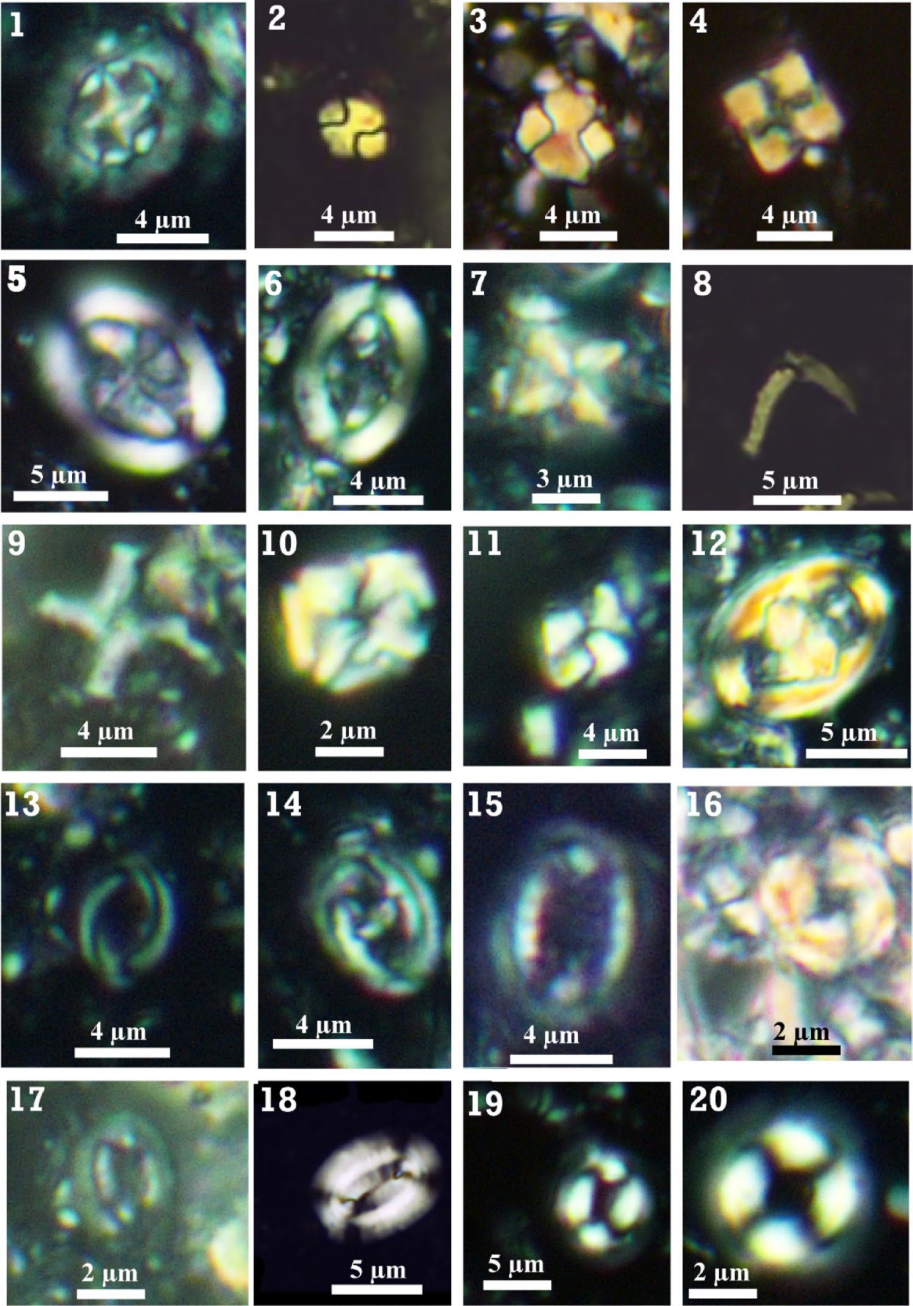
Photomicrographs for the nannofossil most representative taxa: **1-** *Prediscosphaera cretacea*, sample 20 m, Zone UC20, **2:3-** *Calculites obscurus*, sample 120 m, Zone UC18, **4-** *Uniplanarius gothicus*, sample 125 m, Zone UC18, **5:6-** *Arkhangelskiella cymbiformis*, sample 70 m, Subzone UC20b, **7-** *Micula staurophora*, sample 40 m, Subzone UC20b, **8-** *Ceratolithoides kamptneri*, sample 13 m, Subzone UC20c, **9-** *Micula prinsii*, sample 25 m, Subzone UC20d, **10:11-** *Micula swastika*, sample 63 m, Subzone UC20c, **12-** *Zeugrhabdotus embergeri*, sample 70 m, Subzone UC20b, **13:14-** *Placozygus spiralis*, sample 117 m, Zone UC18, **15-** *Cribrosphaerella ehrenbergii*, sample 63 m, Subzone UC20c, **16-** *Cribrocorona gallica*, sample 90 m, Subzone UC20a, **17-** *Biscutum constans*, sample 127 m, Zone UC18, **18-** *Elipsolithus macellus*, sample 7 m, Zone NP4, **19-** *Coccolithus pelagicus*, sample 5 m, Zone NP4, **20**- *Ericsonia subpertusus*, sample 7 m, Zone NP4.

**Fig. 4:**
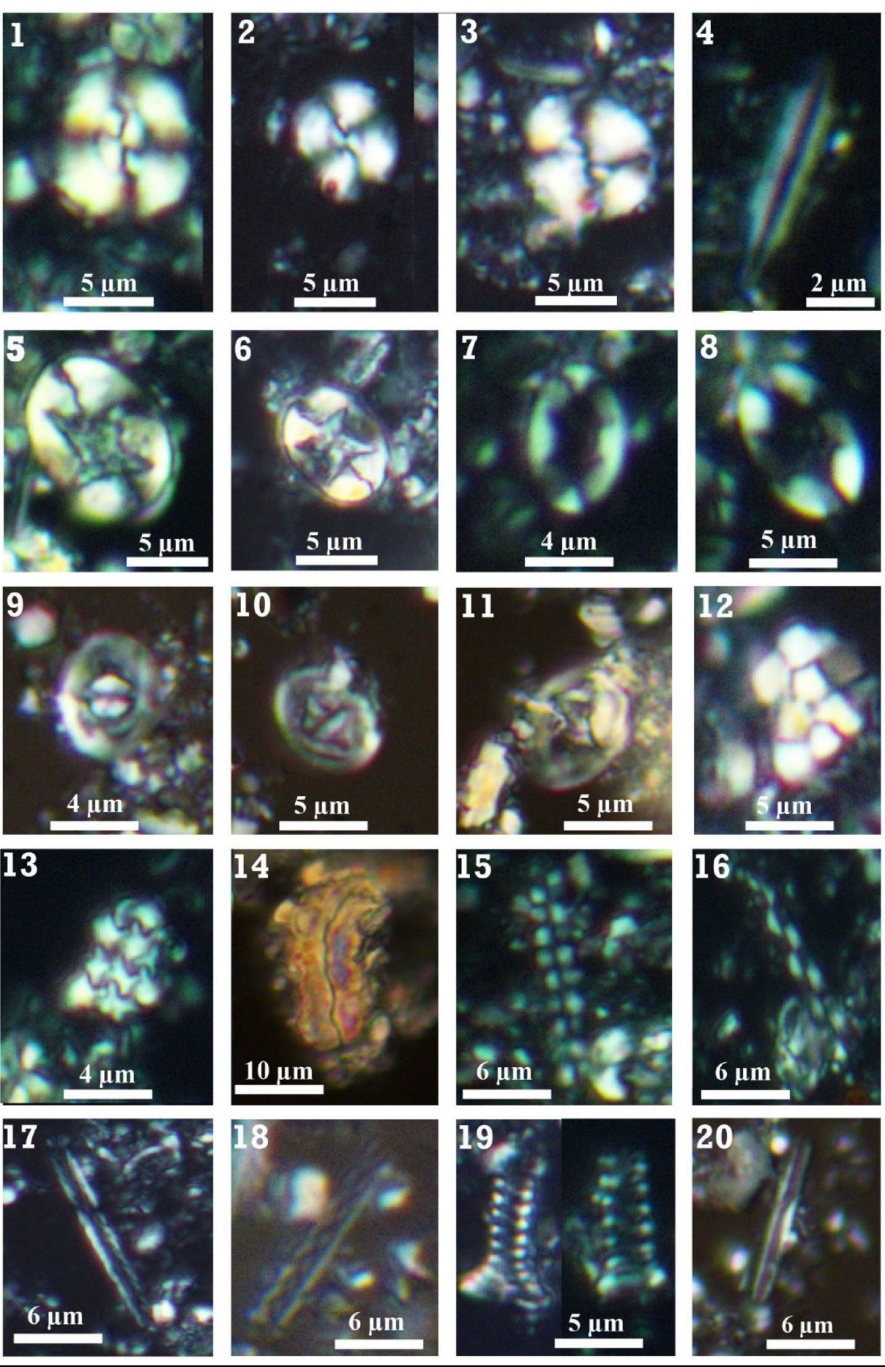
Photomicrographs for the nannofossil most representative taxa: **1:3-** *Watznaueria barnesiae*, sample 45 m, Subzone UC20c, **4-** *Lithraphidites quadratus*, sample 37 m, Subzone UC20c, **5:6-** *Eiffellithus turriseiffelii*, sample 43 m, Subzone UC20c, **7:8-** *Eiffellithus gorkae*, sample 53 m, Subzone UC20c, 9:10- *Tranolithus orionatus*, sample 127 m, Zone UC18, **11-** *Reinhardtites levis*, sample 127 m, Zone UC18, **12-** *Cervisiella operculata*, sample 33 m, Subzone UC20c, **13-** *Cervisiella saxea*, sample 25 m, Subzone UC20d, **14-** *Lucianorhabdus cayeuxii*, sample 87 m, Subzone UC20a, **15-** *Microrhabdulus undosus*, sample 20 m, Subzone UC20d, **16:18-** *Microrhabdulus decoratus*, sample 35 m, Subzone UC20c, **19-** *Tetrapodorhabdus decorus*, sample 73 m, Subzone UC20b, **20-** *Lithraphidites carniolensis*, sample 35 m, Subzone UC20c.

For the calcareous nannofossil treatment method, the samples were prepared using the standard “smear slides” technique described by Bown and Young^49^. We investigated and scanned the prepared slide along three random traverses per smear slide in a prefixed area of 4.52 mm to count 300 specimens, sufficient for statistical analysis. Two additional traverses were skimmed to inspect the other rare biostratigraphic markers. The nanno-floral assemblage was easily quantified due to its good preservation status. The calcareous nannofossil taxa were photographed using a digital Nikon camera adjusted on a polarized Nikon microscope with a magnification of 1000X in the central laboratory of the Geology Department, Faculty of Science, Cairo University. The captured nannofossil photos are illustrated in Figs. 3 and 4. The identification of the calcareous nannofossil taxa was implemented based on the Perch-Nielsen^50^ scheme and the published work on the nannotax3 database (https://www.mikrotax.org/Nannotax3/). The identified calcareous nannofossil taxa retrieved from the investigated interval have been tabulated in Appendix 1, and their vertical distribution is illustrated in Appendix 2. Dr. Ramadan M. El-Kahawy has deposited all the accomplished smear slides in his collection, numbered from 1 F to 51 F, and stored for the Geology Department Museum, Cairo University.

Regarding the palynological treatment, the materials of this study were subjected to tap-water washing to eliminate the drilling fluid and other contaminations. About 30 g of ditch cuttings were selected, covering the interval from 2 to 130 m. Using hydrochloric acid (30%), the crushed samples were treated to eliminate carbonate contents. The samples were then washed with distilled water three times, with at least 6 h between each washing step. The samples were then subjected to hydrofluoric acid (50%) overnight for silicate removal, followed by neutralization with distilled water. An approximately 2.0 specific gravity ZnCl_2_ solution was used in a heavy liquid separation. Furthermore, samples were mixed with water, put in an ultrasonic device for 30 s, and then sieved using a 125 μm sieve, 10 μm nylon mesh, respectively. A percentage of the residue was adhered onto two slides using glycerin jelly, and then a 20 × 40 mm slide cover was placed on top.

**Fig. 5:**
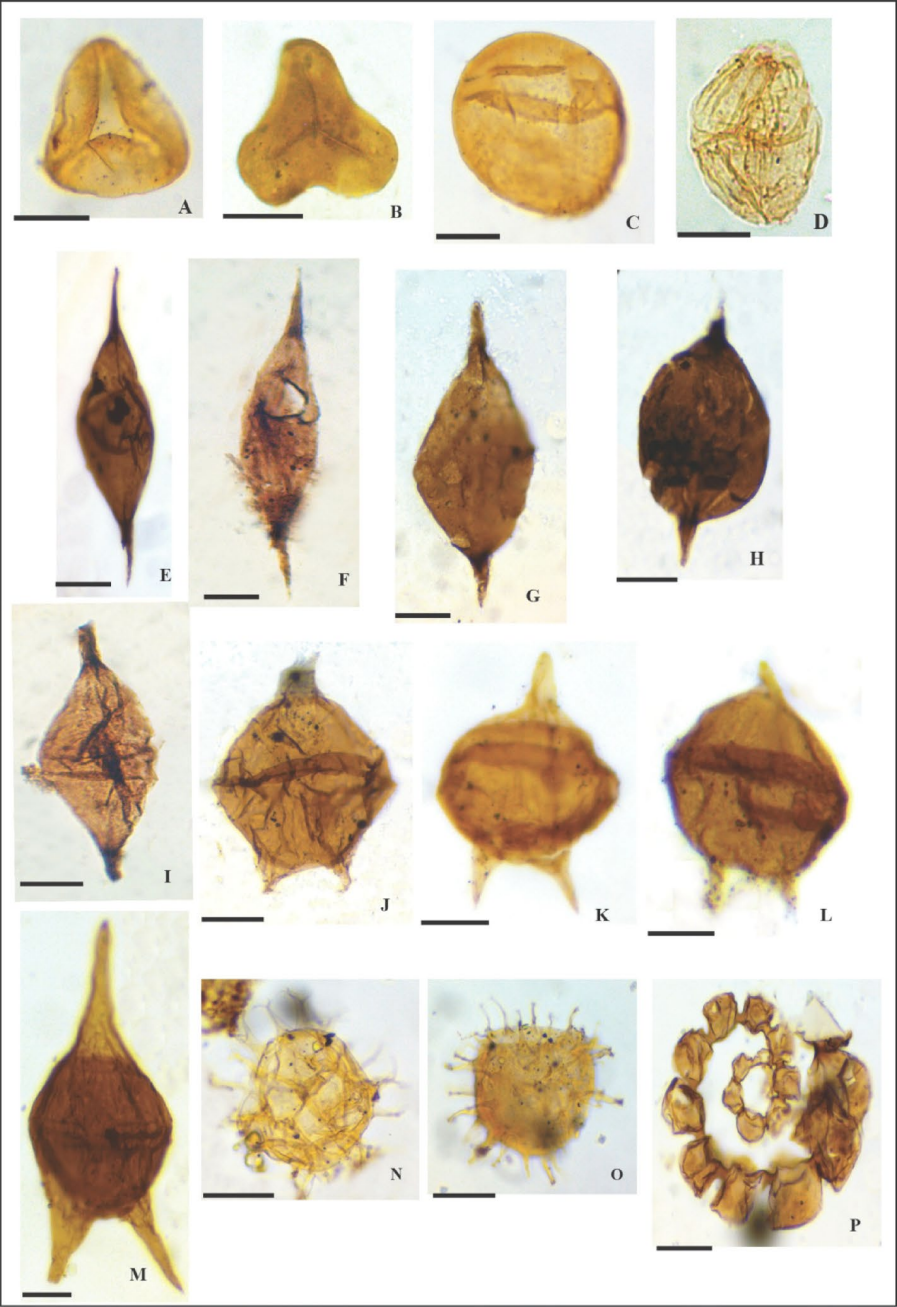
**(A)** *Deltoidospora sp.*,depth 25 m,(microscope coordinates) 42/10, **(B)** *Concavisporites sp.*,depth 33 m,coord. 50/10. **(C)** *Inaperturopollenites sp.*,depth 45 m,coord. 40/11.5. **(D)** *Dinogymnium alberti*,v,coords. 30/11. **(E&F)** *Palaeocystodinium australium*,depth 25 m,coords. 30/8 & 42/10.5. **(G &H)** *Andalusiella gabonensis*,depth 37 m,coord. 33/12.5 &30/11. **(I)** *Andalusiella mauthei*,depth 43 m,coord. 41/11. **(J)** *Cerodinium granulostriatum*,depth 65 m,coord. 34/10. **(K& L)** *Senegalinium bicavatum*,depth 20 m,coords. 30/10 & 42/9. **(M)** *Cerodinium dieblii*,depth 30 m,coord. 40/11. **(N)** *Spiniferites sp.*,depth 23 m,coord. 34/11. **(O)***Downiesphaeridium sp.*,depth 47 m,coord. 32/11. **(P)** *Foraminiferal test lining (FTL)*,depth 30 m,coord. 49/11. Scale bar 30µ.

All the observed palynomorphs and palynofacies from the two slides were counted, and the different palynomorph taxa were identified, with representative forms illustrated in Fig. 5. Five hundred specimens were counted and examined to understand the studied interval’s paleoenvironmental setting using the APP (AOM, Phytoclast, Palynomorph), and Federova ternary diagrams^51,52^, respectively. Moreover, the remarkable stratigraphic changes of palynofacies compositions and palynomorph associations provide significant details of paleoenvironmental assessments.

### Geochemical analysis

The ditch cuttings samples were analyzed for major elemental chemical composition using pXRF version X-MET7000 series of the central laboratory of the Geology Department, Faculty of Science, Cairo University. The X-MET7000 device relies on an Oxford instruments X-ray tube with 40 kV and Rh anode target. The detector type, which converts the light signal into electrical ones, is photodiodes, which is composed of Silicon incorporated with three layers forming a PIN diode arrangement pattern. The device works via heating sample temperature up to 400 °C, and the time is set for readings acquisition at 90 s using soil calibration mode of light element and fundamental parameters (LE FP). About 5 g of the targeted samples were crushed and ground by Agate mortar to be pulverized approximately at 50 μm sediments size. These samples were placed into a crucible covered by a thin layer of polypropylene material. The analyzed elements comprise Al, Ti, Mn, Ca, Sr, and P. These elements are vital for reconstructing the paleoenvironmental variations and paleoproductivity that prevailed during the Late Cretaceous (Maastrichtian)- Early Paleogene (Danian) conditions.

#### Quality control and quality assurance

The sediment samples were validated using the empirical calibration method via certified reference materials (CRMs). The CRM is PACS3 of the marine sediment of the National Research Council Canada. The CRMs preceded and followed each sample measurement process. Consequently, the analytical error ranged between 0% (Sr and Mn) and 1.4% (Al, Si, Ca, Ti, and P). The implemented analytical method has a high degree of precision that can be relied on in interpretations.

### Statistical analysis

The occurrence data (species-sample matrix) was transformed to percentage abundance to weigh their importance for all statistical analyses. The calcareous nannofossil taxa greater than 2% were subjected to statistical analyses. Since the Paleocene-bearing nannofossil taxa have been detected within only three samples, their retrieved taxa were represented by a lower percentage. The two most abundant taxa of the Paleocene, included in the statistical analysis, are *Coccolithus pelagicus* and *Ericsonia subpertusa*. The dataset (Appendix 1) was analyzed using the constrained heat map clustering method of the Ward algorithm with a squared Euclidean similarity index to identify the calcareous nannofossil assemblages. The Cophenetic Correlation Coefficient (CCC) was used to evaluate the resulted dendrograms and how faithfully preserved the distance between points of the original data and ranged between 0.84 and 0.93 for the R and Q-mode, respectively, which means a high value of consistency and goodness of fit^53^. In addition, non-Metric Multidimensional Scaling (nMDS) of the two mode-types (R; species and Q; samples) based on the Bray-Curtis similarity coefficient was used to explore and visualize the relationship among the identified assemblages^54^. The stress values ranged from 0.09 to 0.16 for the R and Q-mode of nMDS, respectively, which means the data was configured with high fitness (Dexter et al., 2018). All analyses were conducted by PAST software V.4.13^55^.

The diversity indices (individual numbers, species richness, Fisher alpha, and dominance) were calculated to investigate the paleoecological conditions. The calcareous nannofossil index of productivity (NIP) and dinoflagellates index of productivity (PI) were calculated based on the formula developed by Eshet and Almogi-Labin^56^.

Furthermore, elemental ratios were used as a proxy for paleoenvironmental reconstruction and paleo-productivity interpretation, along with the relative abundance of the calcareous nannofossil and palynomorphs. All element contents were subjected to aluminum as a normalizer^57^ to avoid the lithological contribution and contamination on the trace and/or major elements concentrations^58^. Accordingly, the paleoproductivty was established based on one of the essential micronutrients, phosphorus (P/Al), and its normalized values to aluminum (detrital element)^59^. The Sr/Al has been used as a paleoproductivty proxy^60–62^, Mn/Al as a paleo-redox proxy to diagnose the paleoxygenation condition^63^, and Ti/Al ratios as a detrital (eolian) proxy^64^.

## Results

### Calcareous nannofossil assemblage, and diversity indices

The investigated section is characterized by high calcareous nannofossil abundance and diversity. Fifty calcareous nannofossil taxa were retrieved from the studied samples spanning the Maastrichtian to Danian stages. From the Maastrichtian age, 38 extinct nannofossil taxa were identified, while only nine taxa and three survivors (originated prior to the boundary and continued into the Danian) were yielded from the Danian.

**Fig. 6:**
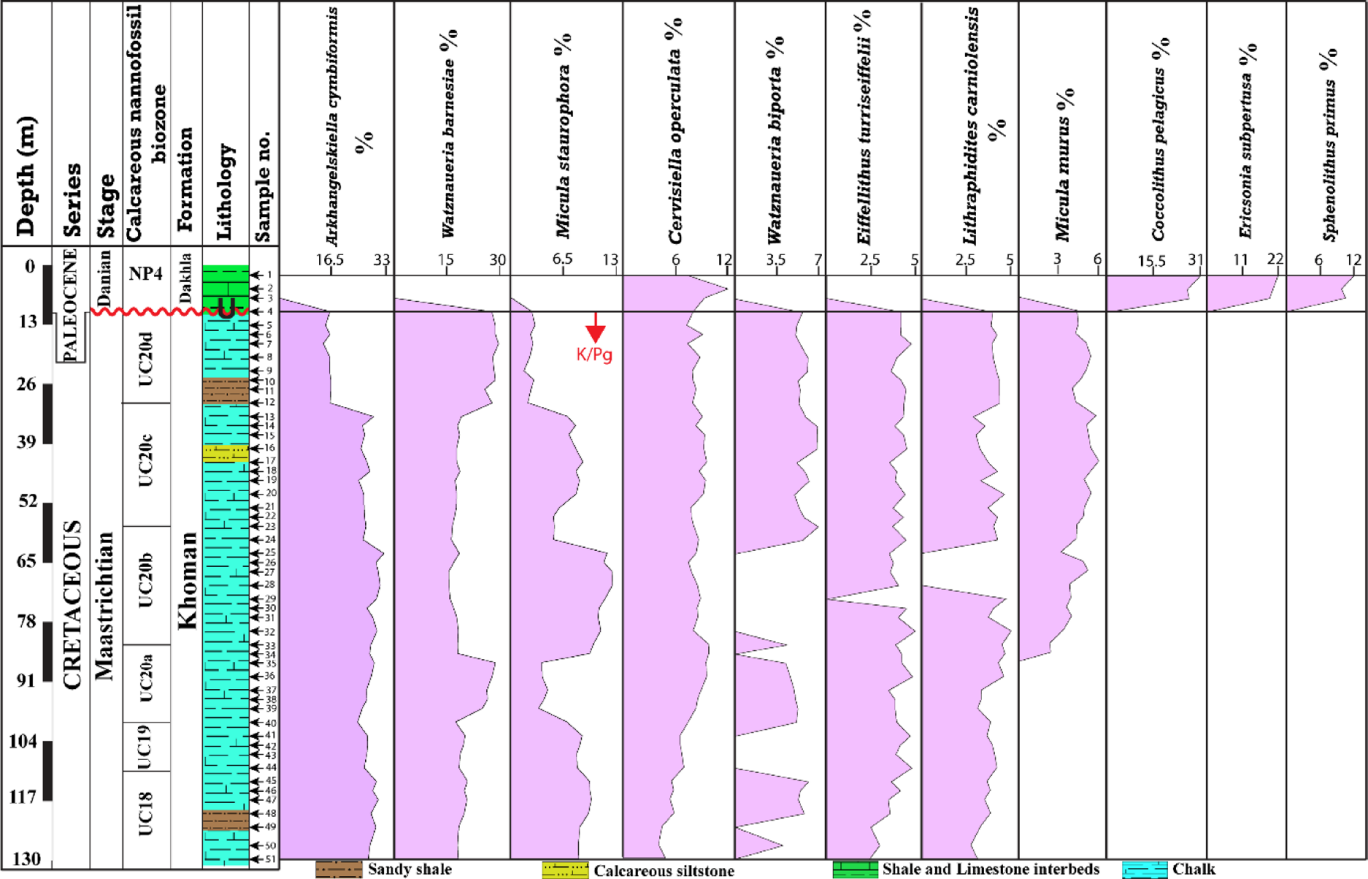
Percentage occurrences of the most abundant calcareous nannofossil taxa across the Maastrichtian and Danian intervals of the B-24 borehole in the Farafra Oasis.

The calcareous nannofossil assemblage from the Maastrichtian interval of the borehole is dominated by eight taxa (Fig. 6). These taxa are represented by *Arkhangelskiella cymbiformis*, *Watznaueria barnesiae*, *Micula staurophora*, *Cervisiella operculata*, *Watznaueria biporta*, *Eiffellithus turriseiffelii*, *Lithraphidites carniolensis*, and *Micula murus*, respectively (Fig. 6). This assemblage consists of about 72% of the total relative abundance of the calcareous nannofossil. The highest relative abundance belongs to *A. cymbiformis*, which accounts for ~ 24% of the Maastrichtian assemblage. Its overall pattern forms a plateau from the base up to depth 33 m, averaging ~ 28%, and declined to be ~ 15.4% mean value in the depths from 30 m to 10 m (Fig. 6). The *W. barnesiae* is the second highest relative abundance of the Maastrichtian assemblage and represents ~ 21.1% of the total assemblage. The taxon distribution vertically averaged 18.49% from the base (130 m) up to a depth of 33 m, except for the depth interval from 97 to 87 m, which attains an average abundance of 27.5%. From the depth of 30 m up to 10 m, interval of the peak, the relative abundance of *W. barnesiae*, averaging 29% and depth of 17 m is the highest (~ 30%) in the whole sequence (Fig. 6). The *M. staurophora* fluctuates through the entire sequence forming three plateaux: from 130 m to 103 m, from 85 m to 63 m, and from 50 m to 33 m (Fig. 6). It is the third highest relative abundance averaging 6.8% and reaches to the maximum at depth 70 m (~ 13%), while the minimum was observed at depth 23 m (1.6%). The *C. operculata*, and *W. biporta* present 7.94%, and 3.56% of the total relative abundance, respectively. *The C. operculata* shows a steadily upward increase to the maximum in the Danian interval, whereas the *W. biporta* displays sporadic occurrences throughout the whole sequence (Fig. 6). Accordingly, the highest relative abundances were observed at depths 5 m (11.3%), and 57 m (~ 6.5%), while the lowest abundances at 127 m (3.9%), and 3.5%, respectively. The *E. turriseiffelii*, and *L. carniolensis* represent 3.14%, and 3.11% of the total relative abundance, and they exhibited an upwards increase with continuous fluctuated occurrences, except at depths 73 m, and four samples occupying depths (63–70 m), respectively. Their relative abundances are the highest at depths 90 (4.2%), and 80 m (4.4%), whereas the lowest were at depths 130 m (1.86%), and 127 m (2.3%), respectively. The relative abundance of the *M. murus* is approximately 2.86% of the total nannofossil assemblage and occurs in the upper part of the Maastrichtian interval, specifically the UC20 Zone. The depth of 43 m represents the highest (~ 5.7%) relative abundance of the *M. murus*, while the lowest is observed at a depth of 83 m (2.4%) (Fig. 6). The Danian interval is represented by three dominant nannofossil taxa: *C. pelagicus*, *E. subpertusa*, and *S. primus* (Fig. 6). Although these three taxa have low relative abundance compared to the total assemblage, they represent a very high percentage of the Danian assemblage. They average approximately 30%, 20.1%, and 10.3%, respectively (Fig. 6).

**Fig. 7:**
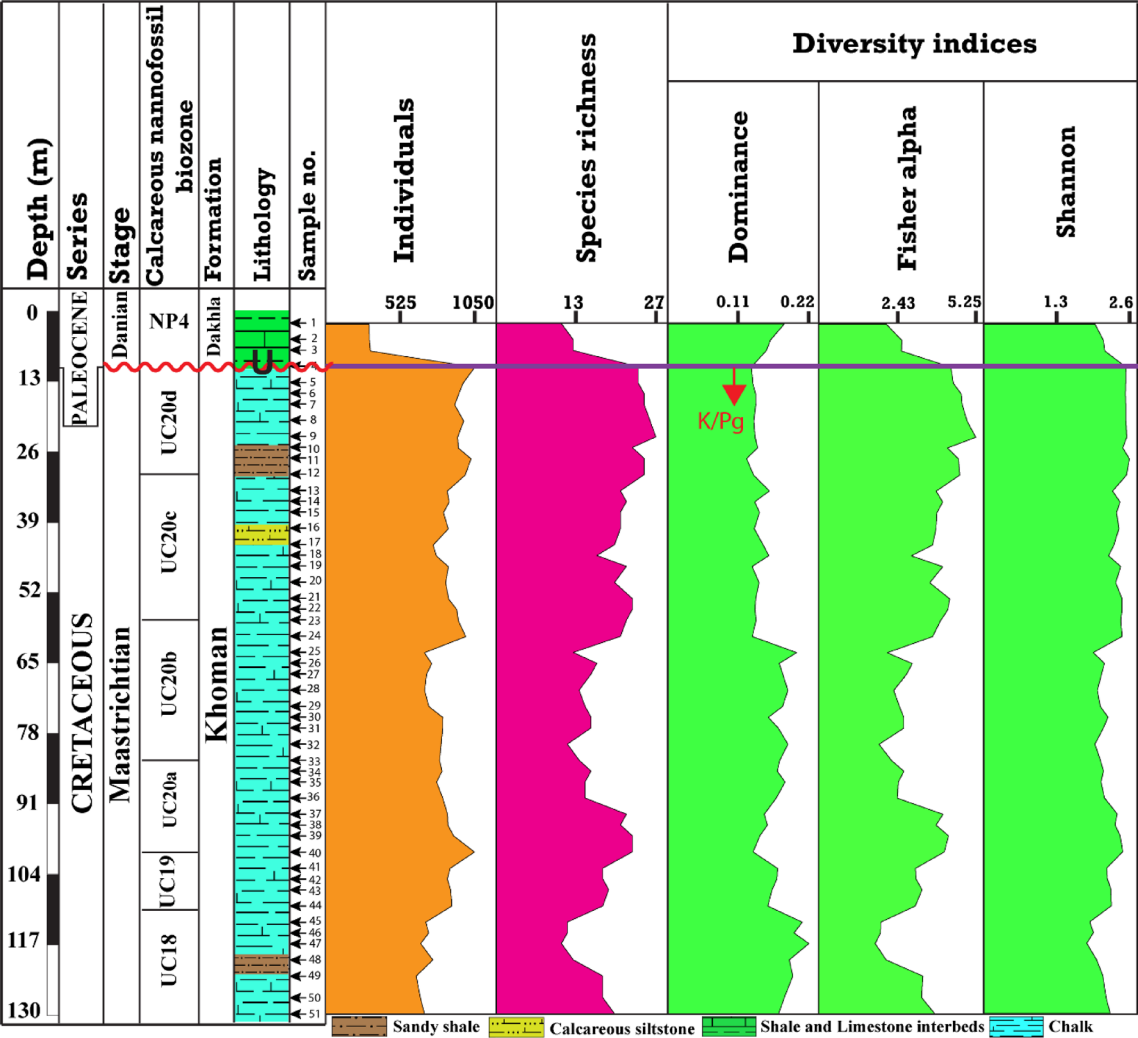
Vertical profile for the calcareous nannofossil individuals, species richness, and diversity indices in the B-24 borehole of the Farafra Oasis.

The calcareous nannofossil assemblage exhibited a highly enriched and diversified community (Fig. 7). The nannofossil abundance of the Maastrichtian stage was the highest at a depth of 10 m, followed by 100 m and 27 m, respectively (Fig. 7). Furthermore, the species richness of the upper part of the Maastrichtian interval was more diversified than the lower part of the section. Accordingly, the highest species richness is occupied depths 23 m (*n* = 27), followed by 15 m and 17 m (*n* = 25) (Fig. 7). Notably, nannofossil abundance was lower at the base of the section, gradually increases upward to a peak around 110 m depth, then exhibited steady fluctuations until the uppermost the Maastrichtian, followed by a marked declines at the onset of the Danian interval. In contrast, the species richness trend increases upwards until the uppermost of the Maastrichtian and sharply declines by the latest Danian interval (Fig. 7). The Danian interval shows a sharp decline in the individual numbers and species richness. The highest individuals and species richness is recorded at a depth of 7 m (312 and 13), respectively (Fig. 7). The relationship between the nannofossil species richness and individual numbers was evaluated using diversity indices (Fig. 7). The diversity indices (Fisher alpha, Shannon, and Dominance) possess similar patterns to the species richness curve, which is opposed by the dominance index (Fig. 7). The highest Fisher alpha and Shannon values were recorded from depths 23 m (5.2), and 27 m (~ 2.6), whereas the lowest values were observed from the depth 117 m (~ 1.9 for both indexes), respectively (Fig. 7). The highest dominance value was observed at depth 117 m (0.21), while the lowest value was observed at depth 27 m (0.12).

### Palynomorph analysis

**Fig. 8:**
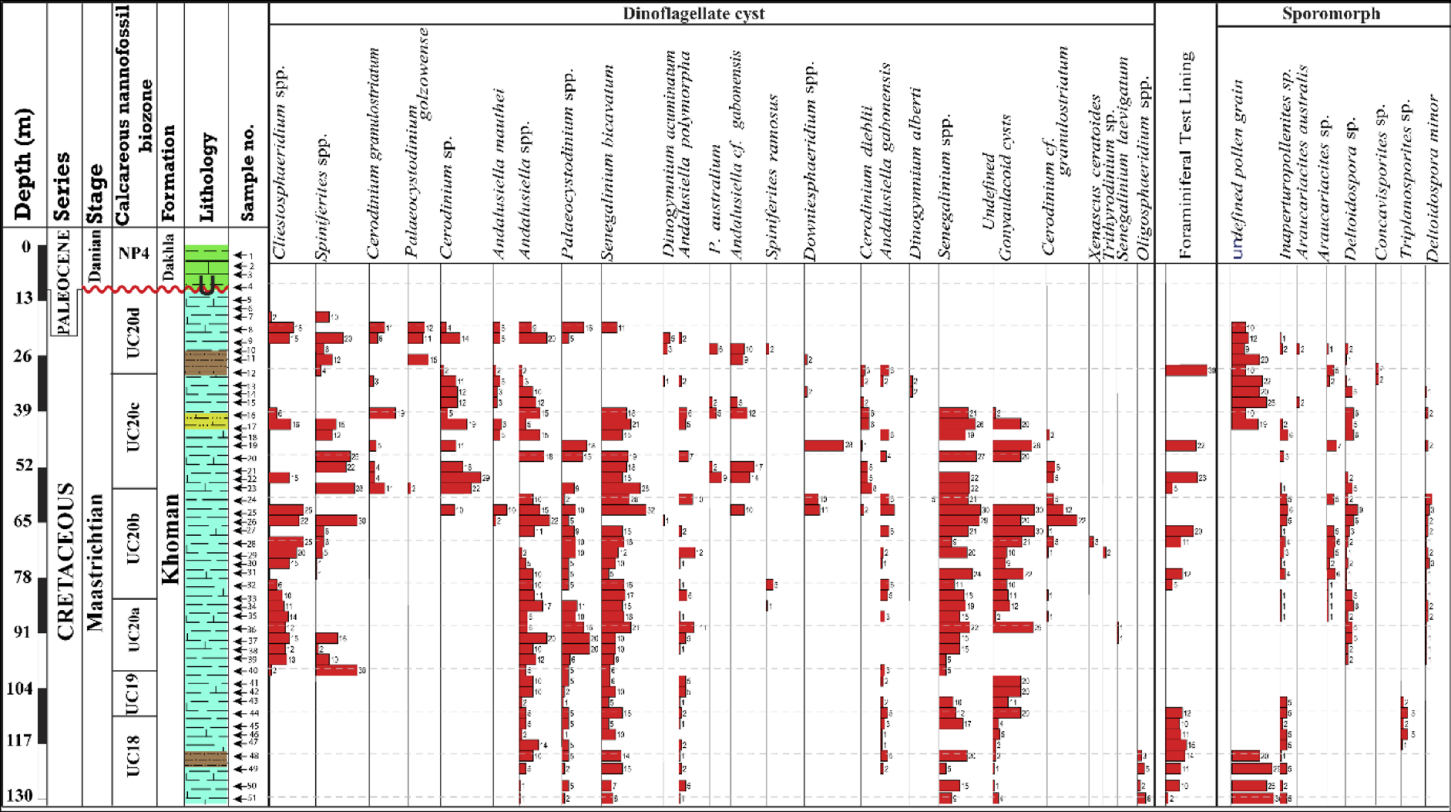
Distribution chart for the relative abundance of the identified palynomorph taxa during the Maastrichtian-Danian interval in the B-24 borehole of the Farafra Oasis.

There are variations in the abundance and diversity of the recovered palynomorph taxa throughout the studied interval; the dinoflagellate cyst assemblage consists of highly abundant and diverse peridinioids that reach up to ~ 69%, whereas the gonyaulacoids are less common (~ 30.6%), and the ptychodiscales are scarce (0.4%) (Appendix 3). The identified peridinioids assemblage is dominated by *Palaeocystodinium australinum*, *P. golzowense*, *Palaeocystodinium* sp., *Cerodinium granulostriatum*, *C. dieblii*, *Cerodinium* spp., *Andalusiella polymorha*, *A. gabonenesis*, *A. mauthei*, *Andalusiella* spp., *Trithyrodinium* sp., *Senegalinium bicavatum*, *S. laevigatum* and *Senegalinium* sp. The peridinioid dinocysts show maximum abundance in the upper (43–23 m) and lowermost parts (130–113 m) of the studied interval whereas, the middle interval (113–63 m) is dominated by gonyaulacoid dinoflagellate cysts that are represented by *Cleistosphaeridium* sp., *Downiesphaeridium* sp., *Spiniferites* sp.,and *Oligosphaeridium* sp. The Ptychodiscales dinoflagellate cyst is represented by the few occurrences of the *Dinogymnium acuminatum*, and *D. albert*. The sporomorph assemblage is present in low proportions, ranging from 0% in many samples to a maximum of 17.24% at 115 m (Appendix 3). It becomes rare in parts of the section and is represented by *Araucariacites australis*, *Araucariacites* sp., *Inaperturopollenites* sp., *Triplanosporites* sp., *Concavisporites* sp., *Deltoidospora minor*, and *Deltoidospora* sp. (Fig. 8).

### Palynofacies assemblages

**Fig. 9:**
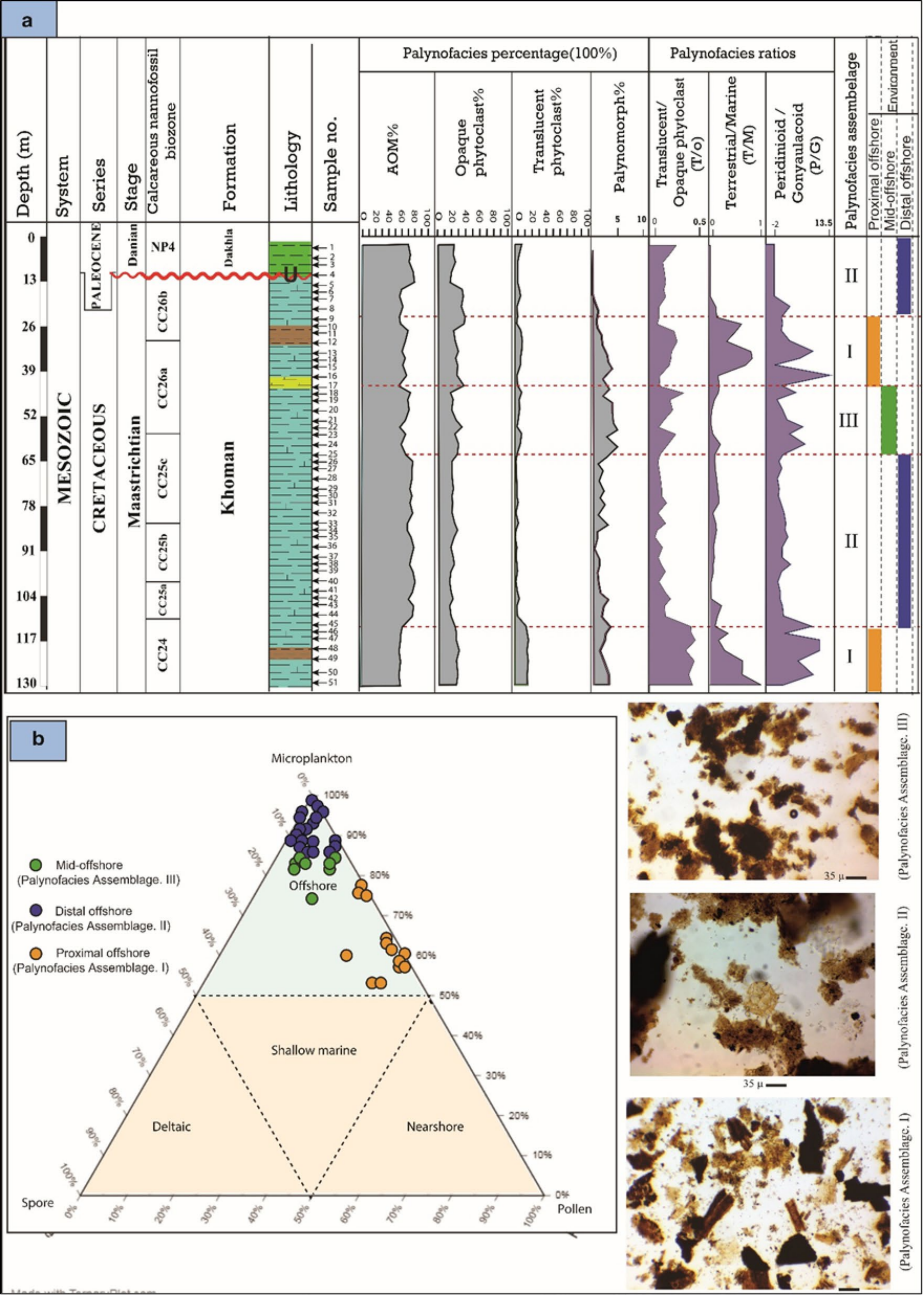
(a) Percentage distribution of kerogen parameters and suggested palynofacies assemblages and their paleoenvironment of the B-24 well. (b) Microplankton, spore, pollen ternary diagram showing the proposed depositional environment of the studied well (after^51^.

Three palynofacies assemblages (I, II, and III) are identified based on the quantitative visual analysis of the total palynofacies groups (based on the counting of 500 particles organic matter) (Fig. 9).

**Palynofacies assemblage I**.

It covers the depth ranges from 130 m to 113 m (samples from 51 to 45) of the lower part of the Khoman Formation and from 43 m to 23 m (samples from 17 to 9) of the upper part of the Khoman Formation. (Fig. 9). It is recognized by a high abundance of terrestrial palynomorphs, while marine palynomorphs (dinoflagellate cysts and foraminiferal test lining) show a remarkable decrease in abundance. The terrestrial over marine ratio (T/M) reaches its maximum value at this interval (Fig. 9). The translucent phytoclasts show relatively high percentages, compared with the rest of the samples (10–15%). The AOM group decreases in abundance, reaching ~ 58%, while the opaque phytoclasts percentage ranges from 30 to 40% of total palynofacies. The large numbers of translucent over opaque phytoclasts ratio (T/O) reflect the large percentage of translucent phytoclasts (Fig. 9).

**Palynofacies assemblage II**.

It occurs from depth intervals 113–63 m (samples from 45 − 25) and from 23–2 m (samples from 9 − 1) (Fig. 9). The high abundance of marine palynomorphs describes this assemblage. The assemblage is dominated by dinoflagellate cysts (gonyaulacoid dinoflagellate cysts), with a notable decline in terrestrial sporomorphs (pollen grains and spores). The AOM group shows a remarkable increase, reaching 80% (of total palynofacies). The translucent phytoclasts percentage is very low (> 2–3%), while the opaque phytoclast is ~ 20% of total palynofacies (Fig. 9).

**Palynofacies assemblage III**.

This covers the depth interval 63–43 m (samples no. 25 − 17) of the Khoman Formation (Fig. 9). It is differentiated by a relatively high percentage of translucent phytoclasts, while AOM and marine palynomorphs (especially gonyaulacoid dinoflagellate cysts) show fairly low percentages compared to assemblage II. The AOM group shows some decrease (~ 66%), and opaque phytoclasts range from 20% to 30%. The percentage of translucent phytoclasts slightly increases, reaching 6% (Fig. 9).

### Temporal variation of elemental proxies

**Fig. 10:**
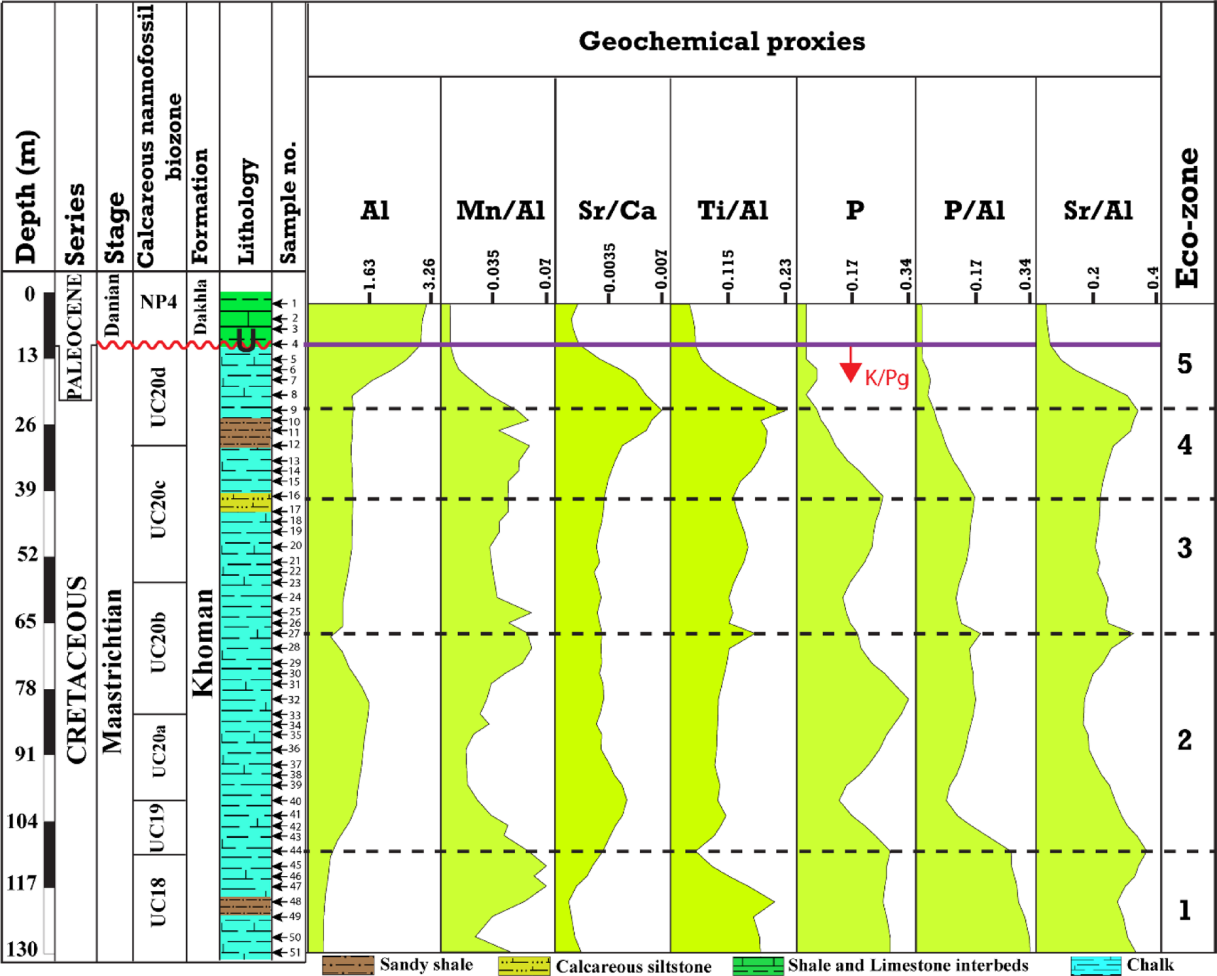
Vertical profile for the measured geochemical ratios with their corresponding eco-zones.

The vertical distributions of the geochemical proxies (Al%, P%, Mn/Al, Ti/Al, Sr/Ca, P/Al, and Sr/Al) of this study are presented in Fig. 10. Five geochemical zones (1–5) occupying depths (from 130 to 110 m; 107 to 67 m; 65 to 40 m; 37 to 23 m; 20 to 2 m) were categorized based on their elemental ratio behaviours (Fig. 10). Interval 1 occupies the lower part of the borehole from depth 130 to 110 m and shows the lowest values for Al% that ranged from 0.85 to 1.47, along with low values of Mn/Al, Ti/Al, and Sr/Ca (ranged from 0.0233 to 0.0714; 0.049–0.204; 0.0038–0.0058, respectively). On the other hand, the P%, P/Al, and Sr/Al values were the highest and showed a declining upward trend. Accordingly, the P%, P/Al, and Sr/Al values ranged from 0.28 to 0.21, 0.329 to 0.119, and 0.272 to 0.362, respectively. The second interval (2) comprises samples from 107 to 67 m and exhibited variable patterns of upward increase and decline trends (Fig. 10). The Al% values ranged from 1.025 to 1.92, Ti/Al from 0.085 to 0.166, Sr/Ca from 0.0054 to 0.0073, Mn/Al from 0.016 to 0.061, P/Al from 0.086 to 0.205, Sr/Al from 0.16 to 0.33, and P% from 0.14 to 0.33 (Fig. 10). Interval 3 involves eleven samples and displayed an overall slightly increasing patterns for most of the used ratios. Accordingly, the Al% values ranged from 1.30 to 1.54, Mn/Al from 0.033 to 0.061, Sr/Ca from 0.0055 to 0.0059, Ti/Al from 0.114 to 0.152, P/Al from 0.114 to 0.168, Sr/Al from 0.21 to 0.24, and P% from 0.15 to 0.26 (Fig. 10). The interval 4 consists of seven samples and displayed upward increase for the detrital elements and ratios indicators, whereas the P% and P/Al values were upward declining. The Al%, Mn/Al, Ti/Al, Sr/Al, and Sr/Ca values were ranged from 1.5 to 1.55, 0.045 to 0.06, 0.13 to 0.22, 0.22 to 0.33, and 0.006 to 0.009, respectively (Fig. 10). On the other hand, the P%, and P/Al ranged from 0.08 to 0.22, and 0.05 to 0.14 (Fig. 10). Interval 5 comprises five samples of the latest Maastrichtian and three of the Danian samples, where all of the illustrated elements and ratios declined except Al% which leapt sharply to the highest measured value in the Danian samples. Accordingly, the Al% ranged from 1.52 to 3.26, whereas the Mn/Al, Ti/Al, Sr/Al, P/Al, Sr/Ca, and P% ranged from 0.006 to 0.03, 0.03 to 0.16, 0.05 to 0.3, 0.01 to 0.04, 0.004 to 0.008, and 0.05 to 0.08 (Fig. 10).

### Paleoproductivity, paleoclimate, and paleofertility taxa analysis

**Fig. 11:**
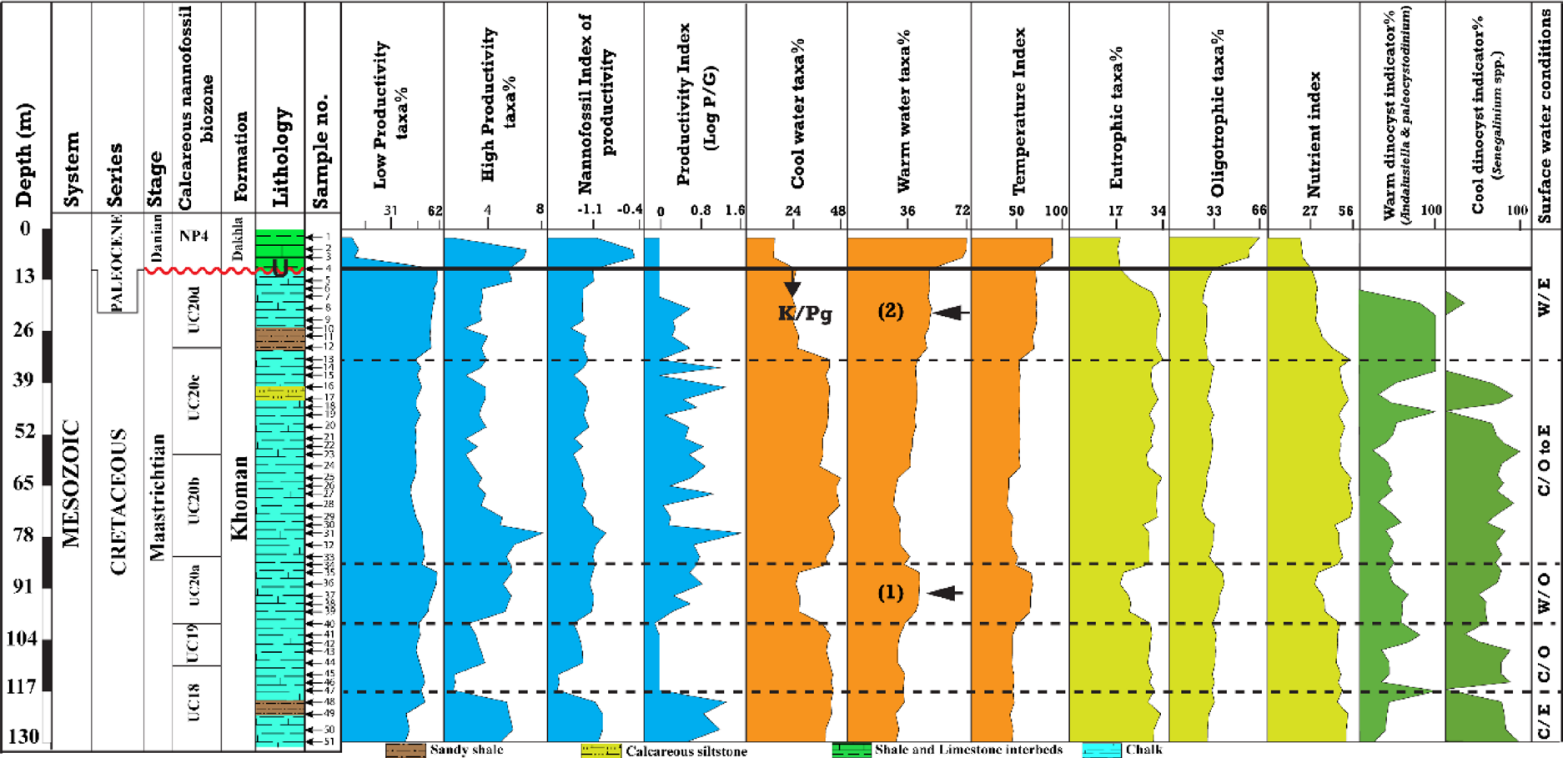
The calcareous nannofossil and dinoflagellate cyst paleoproductivity, paleoclimatic, and paleofertility of the surface water conditions of the B-24 borehole. n***ote***: W- warm, C- cool, E- eutrophic, and O- oligotrophic.

The paleoproductivity of the Maastrichtian has been reconstructed using calcareous nannofossil and palynomorph indicators. The low and high-productivity calcareous nannofossil taxa are introduced in Table 1 and illustrated in Fig. 11. The low productivity taxa increase up to 62.2% at 87 m depth, slightly decrease toward 33 m, then rise again, peaking at ~ 63% at 13 m (Fig. 11). Notably, low-productivity taxa drop sharply from a 52.5% in the Maastrichtian to 9% in the Danian, while high-productivity taxa remain consistently lower in abundance. Accordingly, the highest value in the Maastrichtian interval was 5.7% at depth 13 m, whereas the lowest value was 0% at depths 113–120 m, averaging 2.7%. The Danian interval showed the highest proportion of the high-productivity taxa at a depth of 5 m (6.8%) and averaged 4.9%. Furthermore, the NIP and palynomorphs productivity index (PI) values exhibited overall low productivity conditions in the Maastrichtian period, while a high-productivity trend (up to 0.6) was observed for the Danian interval (Fig. 11). The PI values fluctuated along the Maastrichtian interval, with three high zones: 120 m (1.26) in the lower part, 77 m (1.55) in the middle (the highest value), and 35 m (1.13) in the upper part (Fig. 11).

**Table 1:** List of the calcareous nannofossil ecological indicators used in the present study.

Age	Species	Warm-water taxa	Cool-water taxa	Low productivity	High productivity	Oligotrophic taxa	Meso-Eutrophic taxa	Preservation indicators	References
Danian	***C. pelagicus***	✔			✔	✔			**108**,** 121**
	***C. tenuis***		✔				✔		**141**
	***C. primus***		✔				✔		**141**
	***N. modestus***						✔		**124**
	***E. subpertusa***	✔				✔			**123**,** 126**
	***Chiasmolithus*** **spp.**		✔				✔		**121**,** 142**
	***S. primus***	✔				✔			**142**
Maastrichitian	***C. saxes***	✔			✔		✔		**46**
	***C. operculata***	✔		✔					**26**,** 143**
	***M. murus***	✔							**108**,** 109**,** 113**
	***U. sissinghii***	✔							**25**
	***Z. embergeri***	✔			✔		✔		**121**,** 26**,** 143**
	***M. prinsii***	✔							**144**
	***W. barnesiae***	✔		✔		✔		✔	**109**,** 112**,** 113**,** 121**,** 145**
	***L. carniolensis***	✔		✔		✔			**46**
	***C. kamptneri***	✔							**146**
	***Z. sigmoides***		✔		✔		✔		**119**
	***E. turriseiffelii***		✔	✔		✔			**108**,** 46**
	***N. frequens***		✔						**25**,** 142**
	***A. cymbiformis***		✔				✔		**109**,** 25**
	***M. staurophora***		✔	✔				✔	**46**,** 83**,** 108**,** 109**
	***E. gorkae***		✔	✔		✔			**25**,** 109**
	***E. parallelus***		✔	✔					
	***M. decoratus***		✔	✔		✔			**28**,** 84**,** 112**
	***P. stoveri***		✔						**109**
	***R. levis***		✔						**25**,** 84**,** 109**
	***L. quadratus***		✔	✔		✔			**26**,** 28**,** 46**
	***C. ehrenbergii***					✔			**117**,** 147**
	***P. cretacea***			✔		✔			**46**,** 109**
	***M. undosus***			✔		✔			**28**
	***B. constans***				✔		✔		**112**,** 148**

Based on the recognized calcareous nannofossil affinities and their sea-surface temperature (SST), the paleoclimatic condition of the Maastrichtian- Danian interval has been assessed (Table 1; Fig. 11). Generally, the lower part of the section is characterized by cooler conditions, and the cool-water indicators are averaging 42.1%, while the warm indicators 32% (Fig. 11). Following that, an episode of warmer conditions (depths from 97 to 87 m) within the UC20a Subzone and characterized by mean value of ~ 43% and 26.7% for the warm and cool water indicators, respectively (Fig. 11). On the other hand, the depth interval from 85 m to 33 m averaged 42.4% cool-water taxa, and 36.4% of the warm-water nannofossil taxa. Interestingly, the overall trend of the relative abundance of the cool dinoflagellate cyst representative (*Senegalinium* spp.) shows high percentages and reaches a maximum of ~ 90% (55 m) (Fig. 11). The depth interval corresponding to episode 2 (Fig. 11), is characterized by the dominance of nannofossil warm-water taxa, comprising an average of 49%, whereas cool-water taxa represent 25.1%. It is also characterized by a high abundance of warm-water dinoflagellate cyst indicators (e.g., *Palaeocystodinium* spp., *Andalusiella* spp^58,119^.,, where their relative abundance averaged > 95% (Fig. 11). The Danian (at depth 5 m), the mean value of warm-water nannofossil taxa accounted for 72%, while was 14.3% for the cool-water taxa.

The paleo-fertility conditions were assessed using the relative abundance of the oligotrophic and eutrophic taxa, as well as the nutrient index (NI) (Table 1; Fig. 11). The lowermost part (from 130 to 123 m) of the section is characterized by eutrophic taxa% > oligotrophic taxa%, averaging 30.2%, and 28%, respectively. From depth 120 m up to 75 m, the oligotrophic taxa% > the eutrophic taxa%, averaging 33.5%, and 25.8%, respectively. The eutrophic taxa% in the interval from 73 m to 63 m is 30.9% > oligotrophic% (26%) (Fig. 11). Following upwards from depth 60 to 10 m, the average oligotrophic taxa% is 35.7%, whereas the eutrophic taxa% is 24% (Fig. 11). Noteworthy that along the episodes 1 and 2, as shown from the NI the eutrophic taxa% is the lowest in expense of the oligotrophic taxa%.

### Multivariate analysis

#### Cluster analysis, and non-metric multidimensional scaling

**Fig. 12:**
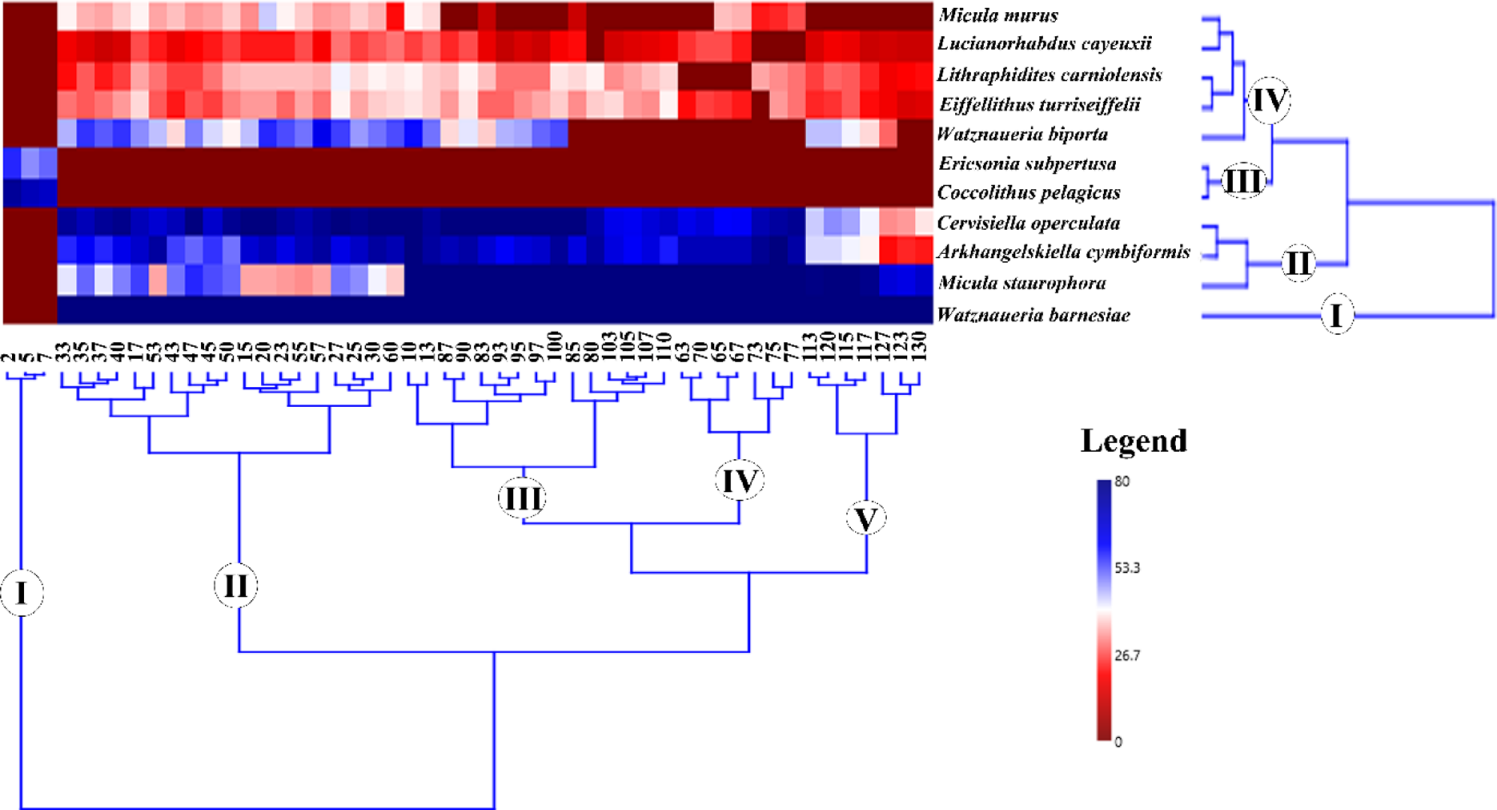
Two-way dendrograms cluster algorithm analysis coupled with colour-coded heatmap for the retrieved nannofossil taxa of the B-24 borehole.

The calcareous nannofossil assemblage was analyzed statistically to discriminate the investigated samples into similar groups (Fig. 12). The cluster analysis of the chosen samples retrieved five clusters (I-V; Fig. 12), each containing characteristic species. Cluster I consists of three samples occupying depths 2, 5, and 7 m. A high abundance of *C. pelagicus* and *E. subpertusa* characterizes this cluster, coupled with low to moderate nannofossil abundance and species richness. Cluster II comprises nineteen samples allocated to the uppermost part of the Maastrichtian Khoman Formation, except samples 10 and 13. These samples are dominated by the occurrence of *W. barnesiae*, *M. staurophora*, *A. cymbiformis*, *W. biporta*, and *C. operculata* and fairly abundant by *M. murus*. Cluster III comprises fifteen samples occupying the depths of the upper lowermost part of the section (from 80 to 110 m). This cluster is dominated by *W. barnesiae*, *M. staurophora*, *A. cymbiformis*, *W. biporta*, and *C. operculata*, while fairly occurrence was observed for both *E. turriseiffelii*, and *L. carniolensis*. Cluster IV consists of only seven samples and occupies the central part of the Maastrichtian Khoman Formation. The cluster is highly dominated by *W. barnesiae*, *M. staurophora*, *A. cymbiformis*, *W. biporta*, and *C. operculata*. Cluster V comprises seven samples that occupy the lowermost part of the borehole from depth of 113 to 130 m. This cluster is highly enriched by two nannofossil taxa *W. barnesiae*, and *M. staurophora*, as well as moderate abundance by *A. cymbiformis and C. operculata* is characterized only by the samples from 113 to 120 m.

**Fig. 13:**
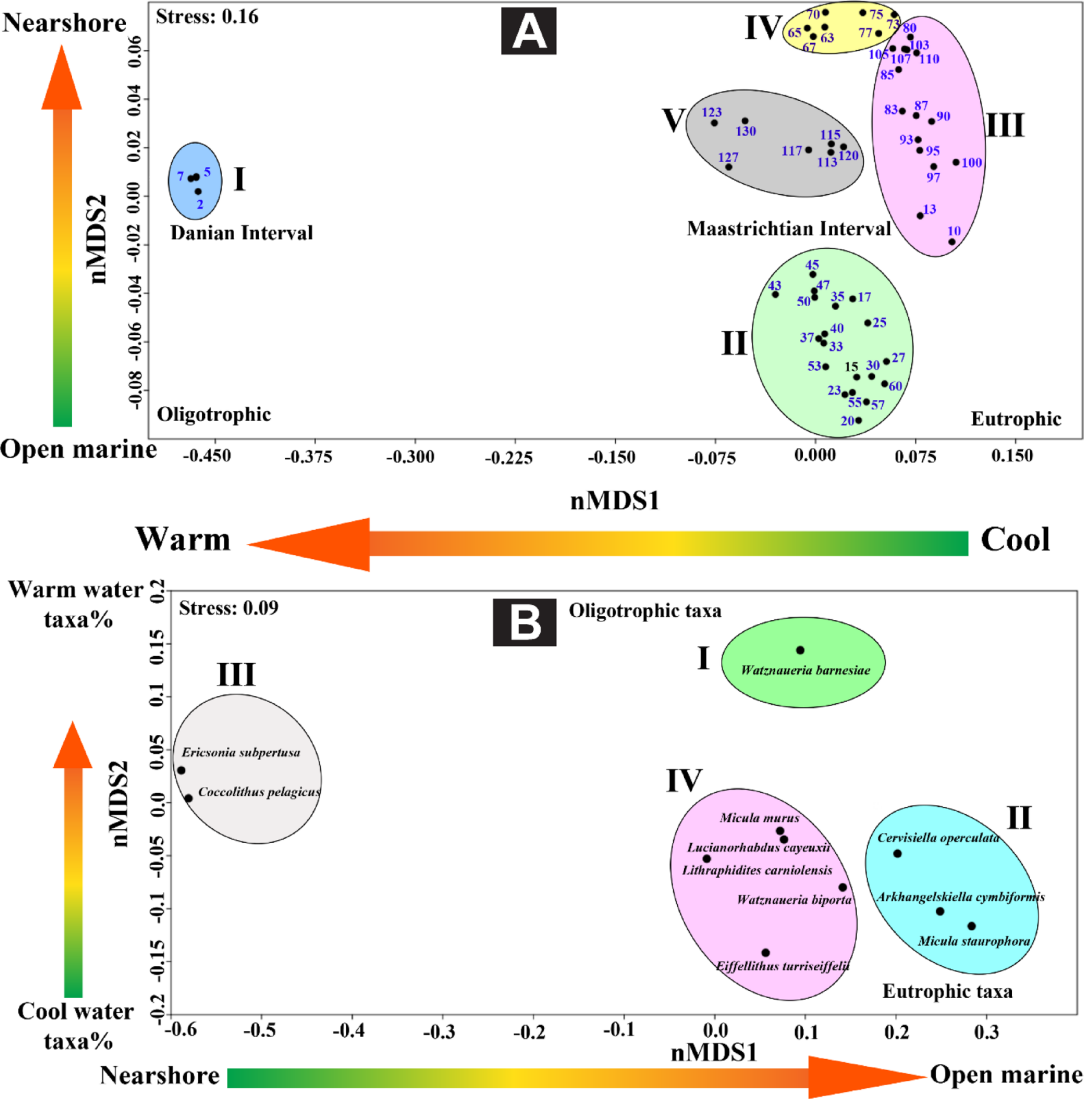
The results of Q (**A**)- and R (**B**)-modes non-metric multidimensional scaling for the calcareous nannofossil taxa of the B-24 borehole.

The ordination of the nMDS analysis demonstrates the retrieved results from the cluster dendrograms, where five groups (I-V) are obtained. The nMDS biplot classified the samples into two main categories: the Maastrichtian samples mostly occupy the right quadrants, whereas the Danian samples are positioned toward the left quadrant (Fig. 13A). Eutrophic conditions characterize the right quadrants, whereas toward the left quadrant, the oligotrophic criterion is more dominant. The Q-mode analysis of the nMDS retrieved five groups that are dominated by nannofossil taxa consistently with the obtained cluster analysis results (i.e., *A. cymbiformis*, and *M. staurophora*). The II and IV groups are dominated by the nannofossil taxa indicators to cool-water conditions, whereas the warm-water taxa indicators dominate group I which is occupied by *W. barnesiae*, and the Danian interval (group III) is occupied by *C. pelagicus* and *E. subpertusa* (Fig. 13B).

## Interpretation and discussion

### Calcareous nannofossil biostratigraphy

The biostratigraphic schemes that we relied on in the present study were for the Mesozoic^65^ and for the Cenozoic^66^ and incorporated with detailed subdivisions nominated by Perch-Nielsen^50^, and Romein^67^. Based on the lowest occurrence (LO) and highest occurrence (HO) of the nannofossil index taxa, four zones (UC17-UC19 and UC20a-d) from the Maastrichtian and only one (NP4) from the Danian have been documented and illustrated in Figs. 3 and 4. The retrieved zones and subzones were discussed below from older to younger as follows:

#### UC18 zone

**Fig. 14:**
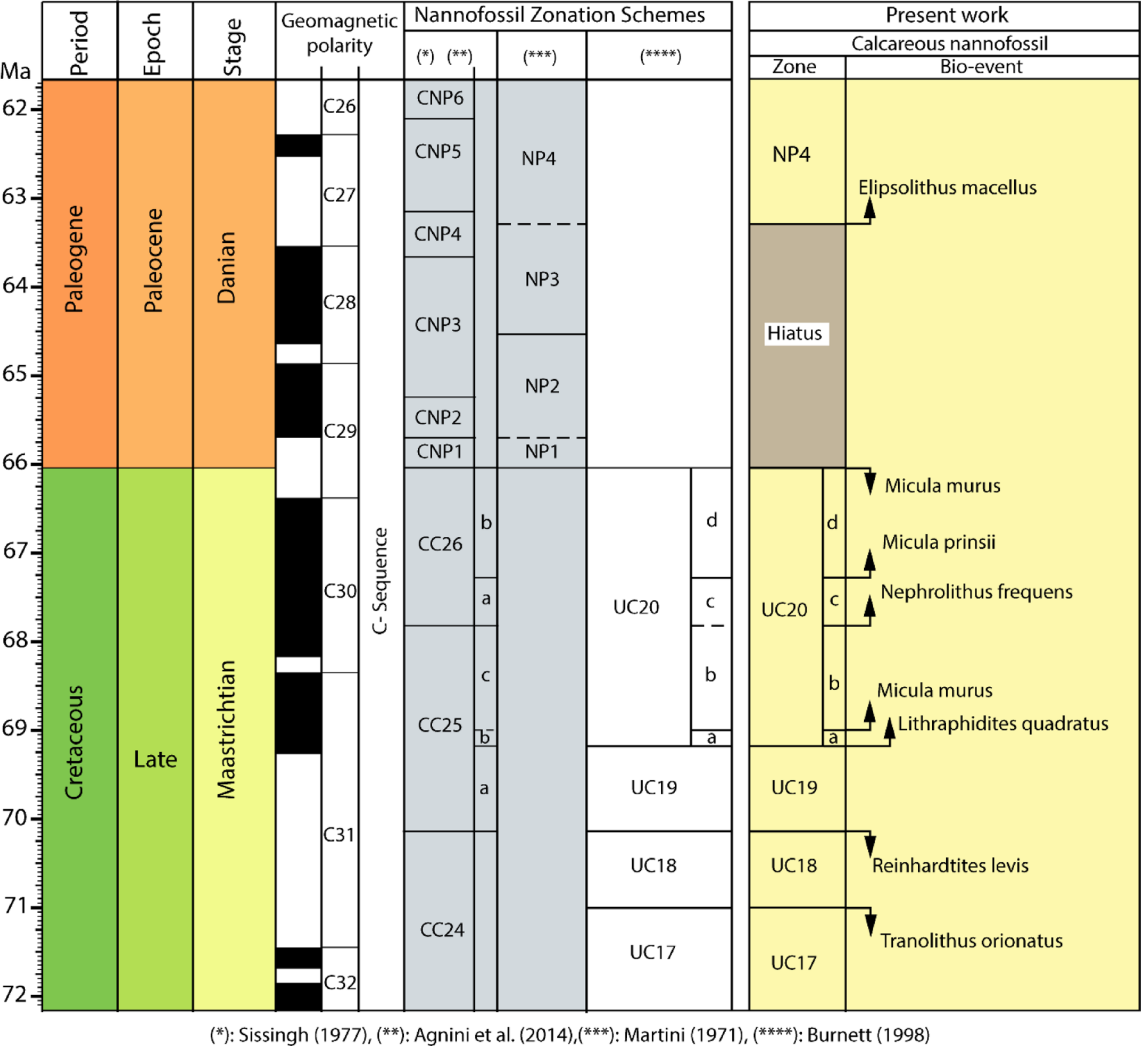
Comparison of the standard nannofossil biozones with the retrieved nannofossil and their bio-events during the Maastrichtian- Danian interval in the Farafra Oasis.

The zone spanned the interval from the HOs of the *Tranolithus orionatus*to the HO of *Reinhardtites levis* (Fig. 14). It is comparable with the CC24 Zone of Sissingh^68^; Perch-Nielsen^50^, and the lowermost part of the *Lithraphidites praequadratus* (NC21) of Roth^69^. The described zone is tentatively placed at a depth of 110 m to 130 m, attains 20 m thick, based on the HO of *R. levis* and HO of *T. orionatus.* The characteristic faunal assemblage of the CC24 Zone comprises the following nannofossil taxa; *W. barnesiae*, *M. staurophora*, *C. operculata*, *Uniplanarius sissinghii*, *R. levis*, *Rhagodiscus angustus*, and *T. orionatus*. This zone consists of the lowest 7 samples of the Khoman Formation. In the southwestern Desert of Egypt, the nominated zone is correlated with Tantawy^70^, where the upper part of the CC24 Zone was barren, and the upper bio-horizon could not be demarcated due to the absence of the marker species *R. levis*. In the present work, the upper zonal boundary is detected via the occurrence of the HO of *R. levis*, whereas the lower zonal boundary is detected via the observed HO of the *T. orionatus* (Fig. 14).

**UC19 Zone**: According to Burnett^65^, the nominated zone spanned the interval from the HO of the *R. levis* to the LO of the *Lithraphidites quadratus*. The described zone is equivalent to the CC25a of Sissinghi^68^, the upper part of NC2 of Roth^69^, and CC25a as well as the lower and middle parts of the CC25b of Perch-Nielsen^71^. The retrieved zone occupies a depth interval from 100 m to 107 m and attains ~ 7 m of the Khoman Formation. The occurrence of the following taxa characterizes the nano-floral assemblage: *Calculites obscurus*, *W. barnesiae*, *M. staurophora*, *Gorkaea pseudanthophorus*, *Microrhabdulus undosus*, *A. cymbiformis* and *Prediscosphaera cretacea* (Appendix 2). The nominated zone is equivalent to the lowermost part of the CC25a Subzone adopted by Perch-Nielsen (1981), the upper part of the NC21 of Roth^69^. In Egypt, Tantawy^70^ identified the upper horizon of this subzone which is represented by the LO of the *A. cymbiformis*, whereas the lower horizon was not observed. Indeed, the lower part of the suggested subzone of Tantawy^70^ is comparable to the nominated subzone (CC25a). In the present work, the UC19 Subzone is demarcated by the LO of the *L. quadratus*, which was first observed at a depth of 100 m (sample no. 40) (Fig. 14).

#### UC20a

This subzone is defined by the LO of the *L. quadratus* and the LO of *M. murus*. The nominated subzone is comparable with the CC25b Subzone of Sissingh^68^, NC22 of Roth^69^, and the lower and middle parts of CC25b of Perch-Nielsen (1985). The zone is dominated by nano-floral assemblage characterized by occurrences of *E. turriseiffelii*, *M. staurophora*, *W. barnesiae*, *Eiffellithus gorkae*, *C. operculata*, *L. carniolensis*, *Placozygus sigmoides*, *Microrhabdulus decorates*, *Retecapsa angustiforata*, *Tetrapodorhabdus decorus*, *Cervisiella saxes*, *Placozygus spiralis*, and *Prolatipatella multicarinata*. This zone is recorded from 87 m to 97 m depth interval of the Khoman Formation. In Egypt, the retrieved zone is equivalent to the recorded CC25b Subzone of Tantawy^70^ from the Dakhla Oasis (Fig. 14).

#### UC 20b

The nominated subzone spanned the interval from the LO of *M. murus* to the LO of the *Nephrolithus frequens*. This subzone is correlated with the CC25c of Sissingh^68^, and the lower part of the NC23 Zone of Roth^69^. On the other hand, this subzone is comparable to the two upper subzones (CC25b and CC25c) of Perch-Nielsen^71^. The nanno-floral assemblage of this interval comprises *W. barnesiae*,* M. murus*,* Micula premolisilvae*, *A. cymbiformis*, *M. staurophora*, *Prediscosphaera stoveri*, *Prediscosphaera cretacea*,* Microrhabdulus undosus*, and *Eiffellithus turriseiffelii*. The subzone is not recorded in southern Egypt at Dakhla Oasis^70^, whereas in the Farafra Oasis (this study), it is recorded at depths from 60 m to 85 m and is 25 m thick (Fig. 14).

#### UC20c

It is defined as an interval subzone between the LO of *N. frequens* and the LO of the *Micula prinsii*. The nominated subzone is equivalent to the CC26a Subzone of Sissingh^68^, the middle part of NC23 Zone of Roth^69^, and CC26a of Perch-Nielsen^71^. The subzone consists of the following nano-floral assemblage *M. murus*, *Retecapsa crenulata*, *E. turriseiffelii*, *Eiffellithus gorkae*, *Micula praemurus*, *Gorkaea pseudanthophorus*, *Biscutum constans*, *Zeugrhabdotus embergeri*, *Placozygus sigmoides*, *L. carniolensis*, and *Lucianorhabdus cayeuxii*. The investigated subzone has occupied depths from 33 m to 57 m and attains 34 m thick. The resultant subzone is equivalent to the recorded subzone CC26a by Tantawy^70^ in southern Egypt (Fig. 14).

#### UC20d

The subzone is defined as an interval between the LO of the *M. prinsii* and the HO of the *M. Murus* or bloom of calcispheres. This subzone is comparable to the upper part of the NC23 Zone of Roth (1978), CC26b Subzone of Sissingh^68^, and CC26b Subzone of Perch-Nielsen^50,71^. It is characterized by nannofossil assemblage comprised *W. barnesiae*, *W. biporta*, *M. murus*, *M. prinsii*, *N. frequens*, *A. cymbiformis*, *M. staurophora*, *E. turriseiffelii*, *C. operculata*, *Zeugrhabdotus embergeri*, *Cribrosphaerella daniae*, *Placozygus spiralis*, *L. carniolensis*, and *Ceratolithoides kamptneri*. The retrieved subzone is penetrated at the depth interval from 10 m to 30 m and attains 20 m thick. It was recorded by Bazeen et al.^72^ in the Abu Minqar area, Faris et al.^73^ in the Farfara district, and Tantawy^70^ in the Dakhla Oasis. On the other hand, it was not observed in southern Egypt by Faris et al.^73^, and Tantawy et al.^74^.

#### NP4 zone

It is defined as an interval zone between the LO of the *Ellipsolithus macellus* and the LO of *Fasciculithus typaniformis*. This zone is compatible and consistent with the CP3 Zone of Okada and Bukry^75^, *Ellipsolithus macellus* Zone of Perch-Nielsen^71^, and the whole CNP6 as well as the lower part of the CNP7 of Agnini et al.^76^. The Danian *C. pelagicus*, *E. subpertusa*, *Chiasmolithus danicus*, Toweius pertusus, *Cruciplacolithus primus*, *Cruciplacolithus tenuis*, *Sphenolithus primus*, and *Neochiastozygus modestus* dominate the characteristic nano-floral taxa for this assemblage. The recorded zone is retrieved from the uppermost part of the borehole (10 m thick), which lumps the first three samples. This zone is recorded in several works done on the southwestern Desert of Egypt based on the above-mentioned nominated biomarkers (i.e^74^., at Dakhla area^73^; at Farafra area). In the present work, only the lower bio-horizon, which is represented by the LO of the *E. macellus*, is recorded, while the upper one is not detected (Fig. 14).

### Palynomorph assemblage

The studied interval shows a reasonable percentage (≈ 85%) denoted by a sufficiently representative occurrence of dinoflagellate cysts, especially when compared to the relatively low recovery of sporomorphs (≈ 15%) throughout the studied interval. The recorded dinoflagellate cysts and sporomorph taxa are displayed in the distribution chart of Fig. 8.

The Maastrichtian interval is characterized by the occurrences of four dinoflagellate cyst taxa: *Palaeocystodinium golzowense*, *P. australinum*, *Cerodinium diebelii*, and *Dinogymnium acuminatum* (Fig. 8). The *P. golzowense* was described from Germany by Alberti^77^ and recorded in the Maastrichtian age of Egypt (El Beialy^78^; Makled et al.^79^; Soliman and Slimani^35^; Tahoun and Mohamed^80^. It is also recorded from the Maastrichtian of Morocco^81^ and Germany^82^. This species has also been recorded in the Maastrichtian of Gabon^83^ and Tunisia^84^. Another significant species is *P. australinum*; it was initially recorded in the early Maastrichtian of the Dakhla Formation, Egypt^35,85^, as it was not found in the older Duwi Formation^79^. However, the presence of *P. australinum* suggests the late Campanian–early Maastrichtian^86^. It was also found in Australia, Argentina, Turkey, and Morocco during the early Maastrichtian to early Eocene^87,88^ and in Spain during the late Maastrichtian^89^. The *Cerodinium diebelii* is discovered in southern Spain’s Campanian-Maastrichtian^90^. According to Tahoun and Mohamed^80^, the co-occurrence and relatively high abundances of *Cerodinium diebelii* and *Palaeocystodinium golzowense* indicate an age that is not older than the early Maastrichtian. Another important record in the present study is *Dinogymnium acuminatum*, a globally common marker for Campanian-Maastrichtian strata^78^. According to El Beialy^91^, the highest occurrences of *D. acuminatum* have been used to determine the Campanian-Maastrichtian age of the Khoman Formation in the northwestern Desert of Egypt. The co-occurrence of *Paleocystodinium* and *Senegalinium* supports an age that is not older than the late Campanian age^85^.

### Calcareous nannofossil preservation and paleoecological significance

Overall, the preservation of the nannofossil specimens displayed moderate to good status, whereas the lower part (equivalent to UC19 and UC20a) exhibited moderate preservation due to high detrital influx and low nannofossil abundance compared to the upper part of the Maastrichtian interval. The notable abundance of *M. staurophora* does not indicate dissolution damage, because many other species including those susceptible to dissolution such as *Biscutum constans*, *Prediscosphaera cretacea*, and *Cribrosphaerella ehrenbergii* are well-preserved. Furthermore, high occurrences of *M. staurophora* were also documented by Moshkovitz and Eshet^92^, and Eshet et al.^93^ in samples from the Hor Hahar section, which exhibited good preservation without signs of significant dissolution or overgrowth. Thus, the elevated presence of *M. staurophora* in well-preserved assemblages from the Maastrichtian sediments in the Negev, Egypt, and other locations may reflect a natural increase in abundance^94^, possibly attributed to ecological factors such as high-stress marine environments^93^.

Numerous studies have highlighted the paleoecological importance of various Late Cretaceous nannofossil taxa^14,32,56,95,96^. These investigations elucidate the paleoecological relationships of these taxa, enhancing our comprehension of surface water conditions. The subsequent section addresses the leading paleoecological indices considered in this study.

The cool-water *A. cymbiformis* is predominantly found in most Maastrichtian zones and subzones, except for UC20d (Figs. 12 and 13), and serves as an indicator of a significant global cooling event during the late Maastrichtian^30,97^. Notably, there is a marked reduction in the size of *A. cymbiformis* specimens toward the upper Maastrichtian in the UC20d Subzone, corroborating earlier findings by Faris^98^ and Tantawy^70^ in Egypt, as well as Gardin^99^in Tunisia. This size reduction aligns with a warming phase, as indicated by stable isotope analyses^13^. Our study showed a significant positive correlation (Table 2) as obtained between this species with *M. staurophora* (*r* = 0.86), and *C. ehrenbergii* (*r* = 0.72), and a reasonably positive correlation has been observed with *Z. sigmoides* (*r* = 0.48), denoting their tolerances to cool-water preferences.

**Table 2:** Pearson correlation coefficients for some ecologically sensitive calcareous nannofossil taxa.

	*N*. modestus	Cruciplacolithus spp.	C. danicus	W. barnesiae	A. cymbiformis	M. staurophora	C. ehrenbergii	M. murus	Z. sigmoides	B. constans	*P*. cretacea
***Neochiastozygus modestus***	1										
***Cruciplacolithus*** **spp.**	**0.99**	1									
***Chiasmolithus danicus***	**0.97**	**0.99**	1								
***Watznaueria barnesiae***	−0.65	−0.66	−0.66	1							
***Arkhangelskiella cymbiformis***	−0.77	−0.78	−0.78	**−0.13**	1						
***Micula staurophora***	−0.51	−0.52	−0.52	**−0.21**	**0.86**	1					
***Cribrosphaerella ehrenbergii***	−0.23	−0.23	−0.23	−0.12	**0.72**	**0.67**	1				
***Micula murus***	−0.29	−0.30	−0.30	**0.67**	−0.10	−0.06	**−0.10**	1			
***Zeugrhabdotus sigmoides***	−0.17	−0.17	−0.16	0.16	0.48	0.34	**0.58**	0.13	1		
***Biscutum constans***	−0.32	−0.31	−0.30	0.44	0.34	−0.25	0.29	0.19	**0.64**	1	
***Prediscosphaera cretacea***	−0.62	−0.53	−0.50	−0.23	−0.35	−0.46	−0.62	−0.44	−0.56	0.29	1

*Watznaueria barnesiae* is generally recognized as a warm-water taxon^70,96,97^ that thrives in oligotrophic marine environments^32,100^. In this study, a significant positive correlation (*r* = 0.92) was identified (Table 2) between *W. barnesiae* and the typical warm-water indicator *M. murus*^29,101^. Other taxa associated with oligotrophic marine conditions during the Maastrichtian include *L. carniolensis*, *E. turriseiffelii*, *P. cretacea*, and *Microrhabdulus* spp^56^.

*Micula staurophora* is a cosmopolitan taxon^14,29^ and a significant component of Late Cretaceous assemblages^102^. The paleoecological relevance of this taxon has been extensively discussed^14,29^. Its increased abundance suggests a heightened diagenetic process due to its resistance to dissolution^96^. In the B-24 borehole, the state of preservation is not critically influential, as solution-sensitive taxa (such as *B. constans*, *P. cretacea*, and *E. turriseiffelii*) are well-preserved and abundant. Thus, the high relative abundance of *M. staurophora* may represent a paleoecological signal. A significant positive correlation (*r* = 0.67) was found between *M. staurophora* and the oligotrophic indicator *P. cretacea* (Table 2), suggesting a similar paleoecological affinity for this taxon as an oligotrophic marker. This finding is consistent with the observations of Eshet et al.^93^, Tantawy^70^, and Thibault and Gardin^32^, who proposed that this species may respond to conditions characterized by low nutrients and high stress.

The high-latitude taxa identified in this study are present in low abundances but exhibit notable frequencies at specific stratigraphic levels within the Maastrichtian zones. These taxa include *N. frequens*, *R. levis*, and *T. orionatus*, all of which are commonly found in high frequencies at high-latitude sites but are rare in low-latitude areas, indicating cool surface water conditions^29,97^.

*Cribrosphaerella ehrenbergii* is another cool-water taxon identified in this study and is regarded by Lees^29^ as a cosmopolitan species. Its paleoecological preferences have been debated; while Erba et al.^103^ argue that its high abundance signifies elevated surface water fertility, Linnert et al.^104^ contend it favors lower nutrient levels. Several authors have identified this species as a cool-water indicator^105^. In this study, a positive correlation was observed between *C. ehrenbergii* and the cool-water indicators *M. staurophora* (*r* = 0.67), *A. cymbiformis* (*r* = 0.72), and *Zeugrhabdotus* spp., mainly *Z. sigmoides* (*r* = 0.58), suggesting similar affinities for cool-water conditions (Table 2).

The genus *Zeugrhabdotus* is regarded as an indicator of high surface water fertility, likely indicative of mesotrophic to eutrophic conditions^100,104,106^. In this study, *Z. sigmoides* and *Z. embergeri* were the recorded taxa within this genus. *Z. sigmoides* is typically associated with cool surface water conditions^107,108^, whereas *Z. embergeri* is identified as a warm-water indicator^101,109^. *Biscutum constans* is relatively rare in the investigated Maastrichtian samples compared to *Zeugrhabdotus* spp. (Appendix 2). Many researchers have classified *B. constans* as a high-nutrient indicator, likely reflecting eutrophic conditions^14,56,96^.

During the Danian age, *C. pelagicus* and *E. subpertusa* dominated the calcareous nannofossil assemblage, particularly in the upper part of the studied Danian interval (Appendix 2). Both taxa are interpreted as indicators of oligotrophic and warm-water conditions^110,111^. *Neochiastozygus* spp. (mainly *N. modestus*) shows relatively low abundance in the upper part of the studied Danian strata (Appendix 2). Gibbs et al.^112^ and Self-Trail et al.^113^ proposed a mesotrophic to eutrophic paleoecological preference for *Neochiastozygus* spp. Our findings reveal a significant positive correlation (*r* = 0.8) between *N. modestus* and *Cruciplacolithus* spp. (Table 2), suggesting that these taxa may share a similar paleoecological affinity as indicators of high nutrient levels, possibly reflecting mesotrophic conditions. *Chiasmolithus* spp. is well recognized as a cool-water indicator^114^ and is adapted to mesotrophic to eutrophic marine environments^115^.

### Paleoclimatic perturbation

The surface waters appear to have undergone significant climatic changes during the K/Pg interval compared to the bottom seawater. These changes are reflected in the variations in calcareous nannofossil and dinoflagellate cyst diversity. Generally, the Maastrichtian stage recorded in the B-24 borehole interval is marked by predominantly cool surface waters, interspersed with two warming events linked to meso-eutrophic marine conditions. This is indicated by the frequent presence of cool-water taxa such as *M. staurophora* and *A. cymbiformis*, a moderate occurrence of *Zeugrhabdotus* taxa, and elevated values of eutrophic taxa (Fig. 11). Furthermore, this cooling trend is associated with a marked increase in the relative abundance of the *Senegalinium* spp. dinoflagellate cyst^116,117^, coupled with high nannofossil cool-water assemblage, indicating decreased sea surface temperature. On the other hand, the warming events were recorded within the upper part of the UC20a Zone (samples 40–34) and UC20d Zone (samples 12–4), respectively (Fig. 11). Episode 1 is denoted by the leap abundance of the *W. barnesiae* (∼27%) and *C. operculata* (∼10%) in respect to the cool-water taxa. According to El-Shafeiy et al.^4^, this warming episode was recorded from the eastern desert of Egypt and attributed this event to the phase-1 Deccan volcanism^118,119^, which occurred in the latest part of the CC25b Subzone and prolonged for approximately 1.37 Ma from 68.36 to 66.99 Ma^70,120^. Episode 2, equivalent to LMWE, is marked by a notable increase in the abundance of typical warm-water taxa, including *W. barnesiae* (∼28%) and *M. murus* (∼6%), at the expense of cool-water taxa. Notably, the recent paleotemperature estimation for the LMWE, which is obtained based on integrated and multi-proxy analysis, has led to a warming of 3.9 ± 1.1 °C^17^. This estimate aligns with findings by Barnet et al.^11^, who documented a warming phase of approximately 2.5–5 °C occurring during the late 150–300 kyr preceding the K/Pg boundary. Additionally, eutrophic conditions dominated the assemblage during this period, such as *Cervisiella saxes*, and *Zeugrhabdotus* spp. Palynologically, the two-episode intervals were dominated by the *Andalusiella* spp. and *paleocystodinium* spp., thermophilic dinoflagellate indicators in the tropical-subtropical provinces^25,121^, and high nutrients indicators^122,123^. These patterns indicate a transition toward warmer, eutrophic marine conditions of the surface waters. The second warming episode may correspond to the LMWE, as evidenced by the proliferation of *M. murus*. An increase in the abundance of *M. murus* during the LMWE has been documented at various locations worldwide^14^, suggesting it could serve as a reliable indicator of this event. Overall, the LMWE is a phenomenon recognized globally^15,16,124^, characterized by a temperature increase of approximately 4 °C^10,13^. The main phase of Deccan volcanic activity (phase-2) has been proposed as a potential trigger for the LMWE^22,119,125^. Additionally, the Danian nannofossil zone (NP4) has been deposited under warming and oligotrophic conditions as indicated by the dominance of *C. pelagicus*, and *E. subpertusa*, which is aligned with the reported results by Keller et al.^126^, and Tantawy^70^.

### Paleoproductivity

The calcareous nannofossil and dinoflagellate cyst are significantly used as productivity indicators^4,127,128^. Several dinoflagellate cyst indices have been used to reconstruct paleoproductivity, such as the ratio between heterotrophic and autotrophic taxa^128–131^. Further, the calcareous nannofossils are characterized by their sensitivity to paleoproductivity as inferred from bioindicator taxa that flourished in organic-rich sediments^4^. Accordingly, Eshet and Almogi Labin^56^ developed a logarithmic ratio to reconstruct the paleoproductivity based on high and low productivity taxa indicators. Eshet et al.^93^ and Eshet and Almogi-Labin^56^ estimated the paleoproductivity by examining the relative abundance of some sensitive calcareous nannofossil taxa and comparing their distribution to that of other organisms, including planktonic foraminifera^127^ and organic-walled dinoflagellate cysts^132^. Further, Eshet et al.^132^ investigated the Late Cretaceous paleoproductivty using peridinioid and gonyaulacoid dinoflagellate cyst ratio. Their results indicated that the relative abundances of these taxa fluctuated during high productivity periods, whereas the diversity and abundance of calcareous nannofossils reached their highest levels during low productivity phases. Thunell and Sautter^133^ reported that during high-productivity periods, there was an increase in planktonic foraminiferal occurrences coinciding with a decline in calcareous nannofossil populations. In contrast, Young^134^ proposed that the abundance of calcareous nannofossils increases with high productivity but declines in hyper-eutrophic environments. The Farafra B-24 borehole offers further insights into the correlation between calcareous nannofossil abundance, species diversity, and environmental conditions in central Egypt along with the geochemical perturbations. Notably, Tantawy^70^ validated the relationship between calcareous nannofossils and productivity observed in other Tethyan regions. He concluded that during the late Maastrichtian, high abundance and species richness of calcareous nannofossils were linked to low surface productivity. In the B-24 borehole, there was a significant increase in low-productivity taxa during the Maastrichtian time, while high-productivity taxa were only occasionally present. At the same time, calcareous nannofossils displayed elevated species richness, aligning with the findings of Keller et al.^126^.

**Fig. 15:**
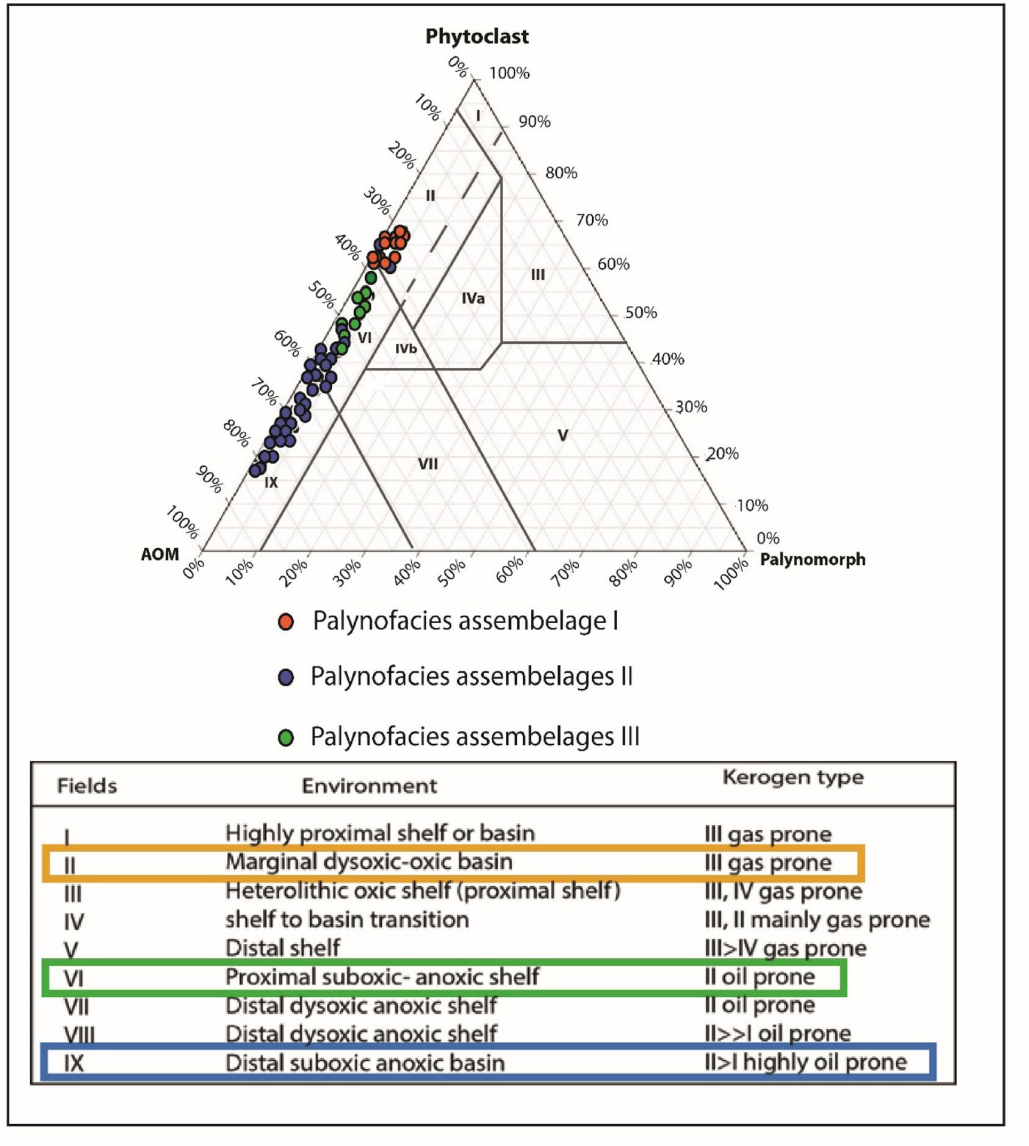
AOM–Phytoclasts–Palynomorphs (APP) ternary diagram showing the oxic/anoxic conditions of the studied samples in the B-24 borehole (after Tyson^52^.

Moreover, the elements that have only one oxidation state, such as Barium (Ba) and Phosphorus (P)^61,135^, and Sr/Al ratio^61,62^, are widely used as paleoproductivty indicators. The elemental ratios support the biotic inferences, where the P%, P/Al, and Sr/Al exhibited high values in the lowermost part of the Maastrichtian interval (Fig. 11). Furthermore, the peridinioid cyst showed high relative abundance, hence a high paleoproductivity as indicated by the high P/G ratio^132^. The decline in productivity toward the end of the Maastrichtian corresponds with trends observed in other areas of the Tethyan Ocean^118,126,136^. This is compatible with the nannofossil, dinoflagellate cyst, and geochemical proxies of the present study, where the high productivity taxa decreased upward, the dinoflagellate cyst ratio fluctuated and decreased, and the P &P/Al trend declined. The patterns of species richness of calcareous nannofossils reflect those reported by Keller et al.^126^, except in the CC25b Subzone, where both abundance and species richness were significantly low. This decline may be due to poor preservation or the presence of a shallow, stressed environment in the Farafra region. During the Danian period, productivity was high, as evidenced by high-productivity taxa within the NP4 Zone. This observation supports Tantawy’s^70^ conclusions, which inferred high surface productivity from the δ13C signature of bulk sediments. On the other hand, the geochemical proxies of the paleoproductivity drivers (P%, P/Al, Sr/Al) claimed the lowest values, whereas the Al% revealed its highest values in the investigated borehole. Thus, several factors address the complexity and discrepancies of interpreting bio-productivity and nutrient dynamics in sedimentary records. Meyers^137^ reported that diagenetic processes play a significant role in preserving organic matter, which carries phosphorus. In contrast, Kowalski and Meyers^138^ suggested that ocean circulation and stratification could enhance nutrient availability in surface waters, increasing productivity but reducing phosphorus retention in sediments. On the other hand, since the Danian interval is characterized by distal suboxic anoxic conditions (see Fig. 15), and the fact that phosphorus is solubilized under reducing conditions^64^, thereby most probably the P is lost from the retained sediments. Furthermore, Schenau et al.^139^ suggested that high productivity is associated with reduced sedimentation rates, facilitating sediment dilution for the paleoproductivty drivers. Accordingly, the Danian interval of the present study suffered from the tectonic activities associated with the Syrian- arc system, which increased the detrital influx causing reduced phosphorus- retained in sediments.

### Paleoenvironmental setting

The biotic proxies are used extensively to reconstruct the paleoenvironmental setting and sea-level changes^5,44^, in particular the Late Cretaceous dinoflagellate cyst has been used as a paleoenvironmental proxy^9^. The paleoenvironmental settings during deposition likely influenced the composition and relative proportions of the palynofacies assemblages. Insights into these environmental conditions were derived from the percentages of amorphous materials, phytoclasts, and palynomorphs (APP) (Fig. 15), as well as microplankton, spores, and pollen, which are illustrated on ternary diagrams. Based on the variations in the percentages and ratios of the palynofacies components and calcareous nannofossil occurrences, three distinct sedimentary settings have been identified: proximal offshore, mid-offshore, and distal offshore (see Fig. 9a and b).

#### Proximal offshore environment

It is characterized by palynofacies assemblage I (Fig. 9a). Notably, there is a decrease in the percentage of the AOM group, accompanied by a sharp decline in marine palynomorphs. This trend is supported by an increase in the T/M ratio, suggesting a slight influence from terrestrial influxes associated with freshwater. The elevated peridinioid to gonyaulacoid (P/G) ratio indicates shallower conditions with some brackish water influence. Peridinioid cysts primarily indicate deposition in estuarine or near-shore lagoonal settings^140^, while gonyaulacoid cysts are associated with relatively open marine environments^141^. Although the samples from palynofacies assemblage I are plotted on the Federova ternary diagram in the offshore field, they fall in the proximal part of the offshore area (Fig. 9b). Notably, the dinoflagellate cyst assemblage predominantly comprises peridinoid cysts, which may suggest increased nutrient availability and productivity often linked to shallower inner neritic settings^142^. However, it is important to recognize that peridinoids can thrive in a wide range of environments, including upwelling zones^122,143^, freshwater-influenced shelves^144^, as well as more open marine systems^145^, and thus their dominance alone does not uniquely signify a proximal offshore environment. Consequently, the integration with the calcareous nannofossil abundance also denoted an upward decrease in diversity indices, suggesting the proximal offshore environment (Figs. 7 and 9a). Additionally, the retrieved palynofacies fall within field II of the APP Tyson diagram^52^, which reflects mainly marginal dysoxic conditions (Fig. 15). Thus, the depositional setting of this interval of the Khoman Formation is compatible with lithological variabilities (coarse clastics) denoting a shallowing upward as suggested by Mahfouz et al.^146^.

#### Distal offshore environment

This environment is classified and reconstructed based on palynofacies assemblage II (Fig. 9). It is characterized by a noticeable upward trend in the abundance of marine palynomorphs, while terrestrial palynomorphs are present in lower quantities, as evidenced by a significant decrease in the T/M ratio. The low P/G ratio suggests a more distal depositional setting as, the high Gonyaulacoid cysts are associated with fully marine, open shelf to outer neritic environments^147^. Additionally, the high occurrence of genus *Spiniferites* is found across a wide range of marine environments; however, several studies have indicated that its abundance tends to increase in more offshore setting^148,149^. Additionally, the AOM group substantially increases, becoming the dominant palynofacies component, and a decline in the T/O ratio further reflects this distal setting. The high proportion of opaque phytoclasts, particularly the equidimensional types, suggests that these materials have been transported considerably from their source area. The samples belonging to palynofacies assemblage II, and of the calcareous nannofossil group II (Fig. 9a) indicate an enhanced marine influence during deposition, pointing to a relatively deeper offshore environment. Furthermore, they predominantly occupy fields IX and VI in the Tyson ternary diagram, suggesting that the condition was suboxic to anoxic basin (Fig. 15).

#### Mid-offshore environment

It is characterized by palynofacies assemblage III, where the AOM group exhibits a relative abundance decline compared to the distal offshore environment. Concurrently, the percentage of marine palynomorphs, particularly peridinioid dinoflagellate cysts, increases as the T/M ratio decreases (Fig. 9a). By analyzing the Federova ternary plot, it indicates that this interval represents a position closer to the transitional offshore environment, along with a high calcareous nannofossil assemblage (Figs. 7, and 9b). The samples from this interval are situated in field VI, suggesting a proximal suboxic to anoxic shelf environment, as inferred from the APP ternary diagram (Fig. 15).

## Conclusion

Integrated calcareous nannofossil and palynological quantitative analysis of the Maastrichtian at Farafra Oasis reveal notable paleoproductivity and paleoclimatic perturbations. The *A. cymbiformis* and *M. staurophora* dominate the calcareous nannofossil assemblage, whereas palynomorphs are dominated by peridinioid dinoflagellate cyst. The overall climatic pattern observed in the investigated section is characterized by cool conditions punctuated by two significant warming episodes. The first episode is marked by a notable decrease in species richness and the presence of warm-water indicators, coinciding with blooms of the nannofossils *W. barnesiae* and *C. operculata*, as well as the dinoflagellate cysts *Andalusiella* and *Palaeocystodinium*. During this period, high-nannofossil productivity taxa were prevalent, yet low-productivity taxa dominated the nannofossil assemblage. The second warming episode aligns with the latest Maastrichtian warming event, during which species diversity remained relatively stable. The paleoproductivty has been reconstructed based on biotic indices (nannofossil and dinoflagellate cyst taxa), as well as geochemical proxy. The lower part of the section revealed high productivity indicators coupled with high P, Sr/Al, and P/Al. The sedimentary setting of the Maastrichtian Khoman Formation was reconstructed to represent proximal offshore in the lowermost and uppermost parts, while the middle part shallowing upward from distal offshore to mid-offshore environments. On the other hand, the Danian Dakhla Formation was diagnosed as representing a distal offshore environment.

## Supplementary Information

Below is the link to the electronic supplementary material.

Supplementary Material 1

Supplementary Material 2

Supplementary Material 3

## Data Availability

Data will be made available from the corresponding author upon request.
